# Magnetism, Conductivity and Spin-Spin Interactions in Layered Hybrid Structure of Anionic Radicals [Ni(dmit)_2_] Alternated by Iron(III) Spin-Crossover Complex [Fe(III)(3-OMe-Sal_2_trien)] and Ferric Moiety Precursors

**DOI:** 10.3390/molecules25214922

**Published:** 2020-10-24

**Authors:** Yuri N. Shvachko, Nataliya G. Spitsyna, Denis V. Starichenko, Vladimir N. Zverev, Leokadiya V. Zorina, Sergey V. Simonov, Maksim A. Blagov, Eduard B. Yagubskii

**Affiliations:** 1 M.N. Miheev Institute of Metal Physics UB RAS, 620108 Yekaterinburg, Russia; starichenko@imp.uran.ru; 2 Institute of Problems of Chemical Physics RAS, 142432 Chernogolovka MD, Russia; spitsina@icp.ac.ru (N.G.S.); max-blagov@mail.ru (M.A.B.); 3 Institute of Solid State Physics RAS, 143432 Chernogolovka MD, Russia; zverev@issp.ac.ru (V.N.Z.); zorina@issp.ac.ru (L.V.Z.); simonovsv@rambler.ru (S.V.S.); 4 Lomonosov Moscow State University, 119991 Moscow, Russia

**Keywords:** hybrid structures, anion radical salts, spin-crossover complexes, organic conductors, SQUID, EPR, magnetic susceptibility, polymorphism, spin-spin interactions

## Abstract

In this study, crystals of the hybrid layered structure, combined with Fe(III) Spin-Crossover (SCO) complexes with metal-dithiolate anionic radicals, and the precursors with nitrate and iodine counterions, are obtained and characterized. [Fe(III)(3-OMe-Sal_2_trien)][Ni(dmit)_2_] (**1**), [Fe(III)(3-OMe-Sal_2_trien)]NO_3_·H_2_O (**2**), [Fe(III)(3-OMe-Sal_2_trien)]I (**3**) (3-OMe-Sal_2_trien = hexadentate N_4_O_2_ Schiff base is the product of the condensation of triethylenetetramine with 3-methoxysalicylaldehyde; H_2_dmit = 2-thioxo-1,3-dithiole-4,5-dithiol). Bulk SCO transition was not achieved in the range 2.0–350 K for all three compounds. Alternatively, the hybrid system (**1**) exhibited irreversible segregation into the spatial fractions of Low-Spin (LS) and High-Spin (HS) phases of the ferric moiety, induced by thermal cycling. Fractioning was studied using both SQUID and EPR methods. Magnetic properties of the LS and HS phases were analyzed in the framework of cooperative interactions with anionic sublattice: Anion radical layers Ni(dmit)_2_ (**1**), and H-bonded chains with NO_3_ and I (**2**,**3**). LS phase of (**1**) exhibited unusual quasi-two-dimensional conductivity related to the Arrhenius mechanism in the anion radical layers, *ρ*_||c_ = 2 × 10^5^ Ohm·cm and *ρ*_⊥c_ = 7 × 10^2^ Ohm·cm at 293 K. Ground spin state of the insulating HS phase was distinctive by ferromagnetically coupled spin pairs of HS Fe^3+^, *S* = 5/2, and metal-dithiolate radicals, *S* = 1/2.

## 1. Introduction

The construction of multifunctional molecular materials, in particular switchable electronic conductors, remains one of the challenges for molecular science, and raises fundamental problems for condensed matter physics [1,2,3,4]. Among synthetic strategies, integration of spin-crossover complexes with metal-bisdithiolene complexes, as redox-active building blocks, gave rise to numerous magnetoactive crystalline materials [5,6,7]. The structures with [M(dmit)_2_]^n−^ complexes in a fractional oxidation state (where (dmit)_2_ is 2-thioxo-1,3-dithiole-4,5-dithiolato, M-3*d* metal ion) are known as low dimensional organic conductors and superconductors [8,9]. In turn, the cationic complexes of the [Fe(sal_2_trien)]^+^ family are well known for the bi-stable configuration of the ligand shell, resulting in the low-spin (LS) or high-spin (HS) states of the Fe(III) ion (*S* = 1/2 and 5/2 respectively) [2,3]. The coexistence of conductivity and SCO properties in a combined hybrid structure opens promising prospective of their mutual synergy. For example, exchange interactions between delocalized spin moments of the conduction electrons and local magnetic moments of HS Fe(III) ions would facilitate ferromagnetic (FM) or antiferromagnetic (AFM) coupling in the SCO sublattice [10,11], which, in turn, may lead to a switchable spin-dependent transport in M(dmit)_2_ sublattice. Therefore, conducting SCO materials are the prospective candidates for molecular spintronics [12,13,14]. Progress toward practical applications has been boosted significantly by observations of light-induced excited spin-states trapping (LIESST) at temperatures below the SCO transition [15,16,17]. Long-living metastable HS phase are ON/OFF switchable by the laser light. In this context, investigation of the magnetic properties and intrinsic spin-spin interactions, and understanding their fundamental mechanisms make an essential influence on the choices of the synthetic approach.

Temperature and laser light are not the exclusive means for generating spin transformations. Organic sublattice, in general, its intrinsic structural changes, as a component of the H-bonded network in the bulk of the hybrid, as well as the electronic structure of the acceptor molecule by itself, are capable of changing spin-states in SCO complexes. A combination of temperature and structure factors of the sublattices gives rise to polymorphic modifications, irreversibilities of the cooperative nature, such as spatially inhomogeneous phases (fractions) withing a single crystal [18,19,20,21,22,23,24,25,26]. Crystallographic identification of phase segregation is a hard task because “spin phases” are not new structures and do not necessarily have different symmetry. Moreover, fractioning is accompanied by an increased amount of disorder. However, magnetic and local probe methods, such as Electron Paramagnetic Resonance EPR, may shed light on the physical properties of the fractions and their relative dynamics [27,28]. With this regard, it does seem important to systematically study the evolution of the inhomogeneous magnetic responses of hybrid SCO structures by magnetic methods.

Hybrid structures of anionic radicals [Ni(dmit)_2_]^−^ alternated by iron(III) SCO sal_2_trien complexes were extensively studied earlier [29,30,31,32,33,34,35,36]. In this work, we report several striking experimental facts regarding the hybrid SCO system [Fe(3-OMe-Sal_2_trien)][Ni(dmit)_2_] and its chemical precursor structures: Electrical conductivity in the anion radical layers of the 1:1 stoichiometric hybrid structure; FM exchange interactions between the anion radicals Ni(dmit)_2_^─^ and magnetic moments of Fe^3+^ of the cations [Fe(3-OMe-Sal_2_trien)]^+^ in HS state; mismatch between factual LS state of the cation in the simple salt [Fe(3-OMe-Sal_2_trien)]I and the angle value between Fe-(Ph-3-OMe) arms (106.55°), formally corresponding to the HS phase. These facts will be explained in the framework of topological phase segregation (fractioning) as an irreversible alternative to the reversible SCO transition in bulk. The alternative arises due to cooperative interactions with the anion radical network.

## 2. Results and Discussion

### 2.1. Crystal Structure

#### 2.1.1. [Fe(III)(3-OMe-Sal_2_trien)][Ni(dmit)_2_] (**1**)

**Figure 1:**
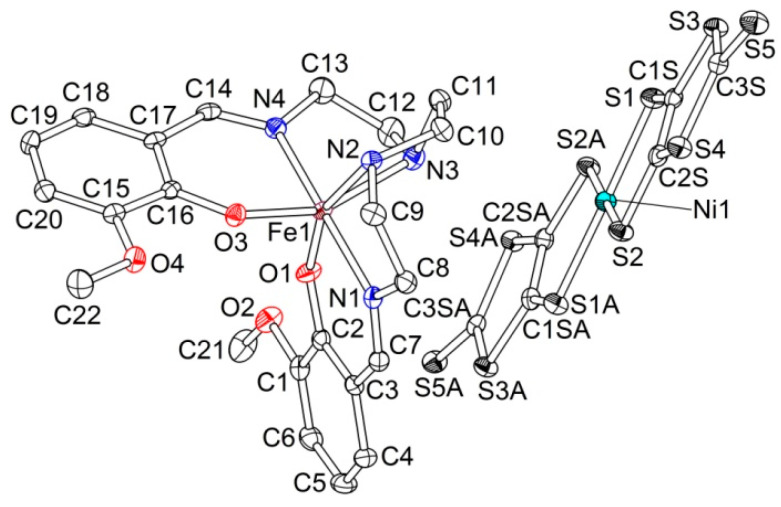
Asymmetric unit in **1** (ORTEP drawing with 50% probability ellipsoids).

**Figure 2:**
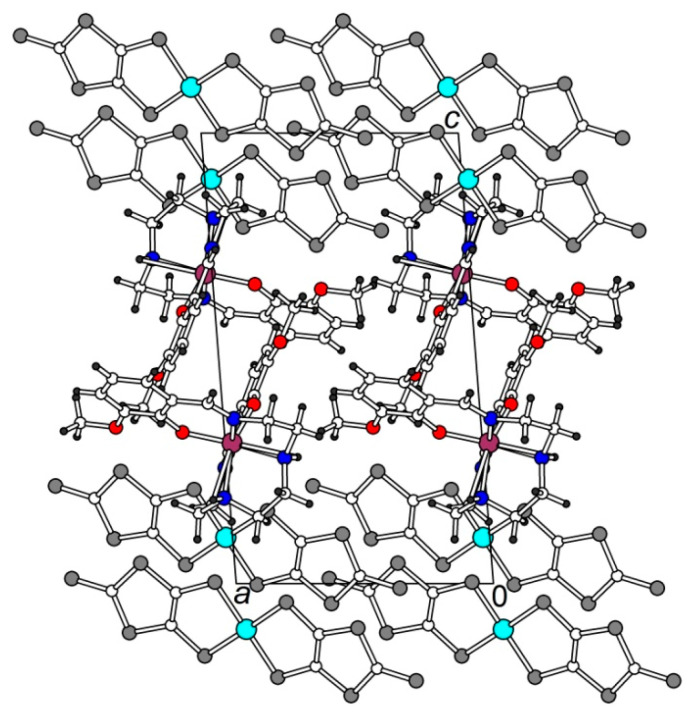
View of the structure **1** along *b*.

Complex **1** has the triclinic *P*-1 symmetry at 120 K, an asymmetric unit contains one cation and one anion, in general, positions (Figure 1). The crystal structure is built of the [Ni(dmit)_2_]^−^ anion radical layers, which alternate with the [Fe(3-OMe-Sal_2_trien)]^+^ cation layers along the *c*-axis (Figure 2).

**Figure 3:**
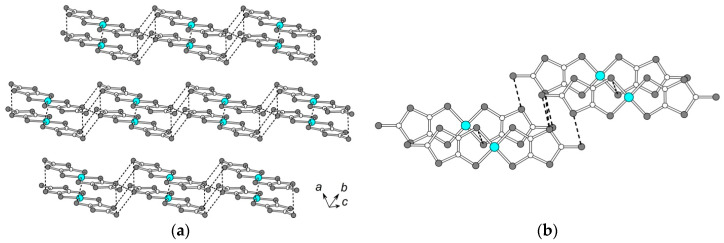
(**a**) The *ab* layer of Ni(dmit)_2_. The S…S contacts < 3.7 Å are shown by dashed lines. (**b**) Overlap mode inside and between the centrosymmetric Ni(dmit)_2_ dimers.

The conducting anion radical layer (Figure 3a) consists of the {[Ni(dmit)_2_]_2_}^2−^ dimers with ring-over-atom intradimer overlap mode (Figure 3b). In spite of the short interplane distance inside the dimer of 3.34(1) Å (×2), only three slightly shortened intradimer S…S contacts of 3.651(1) Å and 3.687(1) Å are found (the S…S contacts less than the sum of van-der Waals radii of sulfur atoms are shown by dashed lines in Figure 3). Adjacent dimers are shifted along the long molecular axis, and there is no interdimer overlapping. However, the shortened S…S interdimer contacts [3.256(1), 3.387(1), 3.521(1), 3.619(1) Å] are observed, and they link the dimers into infinite ribbons along the [1,2,3,4,5,6,7,8,9,10] direction. Interplane distance between the nearest anions from the adjacent ribbons is 3.60(1) Å, and S…S contacts < 3.7 Å are absent.

**Figure 4:**
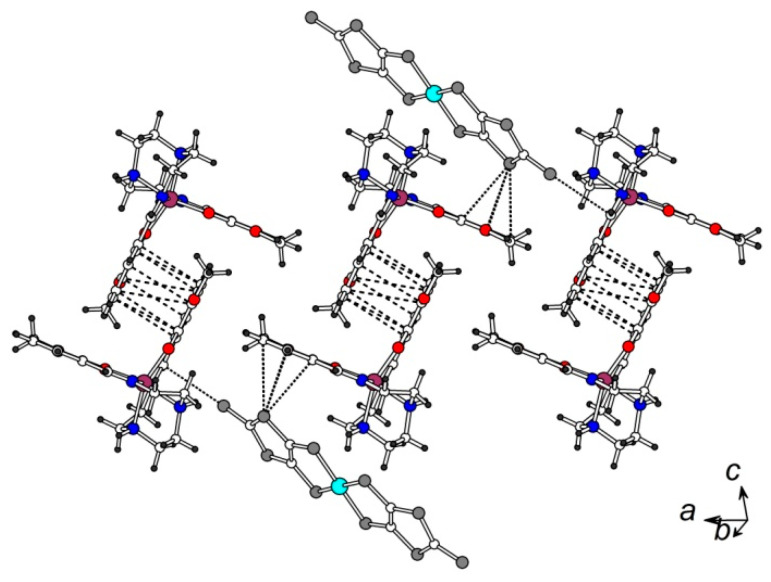
The [Fe(3-MeO-Sal_2_trien)]^+^ chains in the *ab p*lane. The C…C contacts < 3.6 Å in *π*-stacked pairs are shown by dashed lines. The anion…cation S…C contacts are shown by dotted lines.

The magnetic cation layer is composed of the [Fe(3-OMe-Sal_2_trien)]^+^ chains running along the *a*-axis (Figure 4). The cations in the chain are linked into pairs by a *π*-stacking interaction involving two inversionally related salicylidene arms of the nearest cations. The distance between the centroids of two phenyl cycles is 3.472(3) Å, and several intermolecular C…C contacts in the range of 3.376(4)–3.509(5) Å are found. The other salicylidene moiety of the cation participates in *π*…S interaction with the S(3A) atom of the anion, which is also shown in Figure 4. The distance S(3A)…centroid [C(1)–C(6)] is as short as 3.217(2) Å, the S…C contacts are in the range of 3.466(3)–3.560(3) Å.

**Figure 5:**
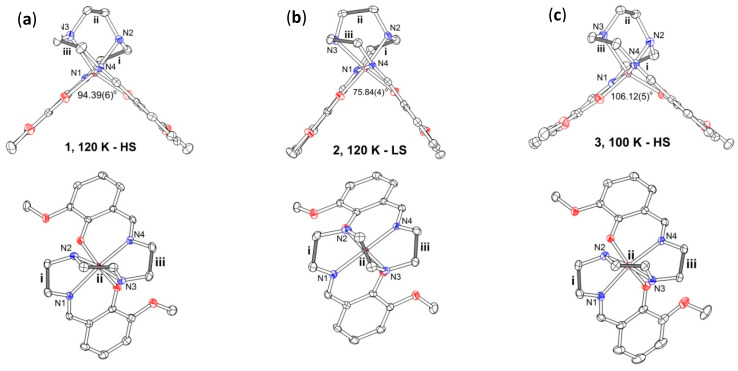
Molecular conformation of the [Fe(III)(3-OMe-Sal_2_trien)]^+^ cation at 120 K in **1** (**a**), **2** (**b**) and at 100 K in **3** (**c**), side (upper row) and top (bottom row) view. The values of *α* angles between the salicylidene groups are given in the figure. The angles between C-C lines of ethylene groups i, ii, and iii are listed in Table 1.

The Fe(III) ion in [Fe(3-OMe-Sal_2_trien)]^+^ is octahedrally coordinated by two oxygen and four nitrogen atoms of the ligand. At 120 K, Fe(III) is in a high-spin state. The latter is confirmed by increased Fe-N and Fe-O bond lengths, as well as significant distortion of the coordination octahedron: O-Fe-N angles are 155.23(8) and 160.47(8)° (Table 1). The obtuse dihedral angle α between two salicylidene groups of 3-OMe-Sal_2_trien of 94.39(6)° also points to a high-spin state of the complex (Figure 5a). Besides, we have shown recently that different conformations of the trien moiety are energetically favorable for HS and LS complexes and can be used as an indicator of spin state of [Fe(III)(sal_2_trien)]^+^ cations [10,25]. In **1**, the central -CH_2_-CH_2_- bond (ii) of the trien is near-normal to the side bonds (i, iii) that is typical for the HS state (Figure 5a and Table 1). We suppose that the absence in complex **1** of a spin-crossover to a low-spin state at cooling down to 120 K is associated with strong *π*…*π* and *π*…S intermolecular interaction of the magnetic cation (involving both salicylidene moieties of the ligand) with the molecular environment (Figure 4) which prevents conformational changes necessary for a spin transition. Exactly the same situation was observed in the isostructural to **1** complex [Fe(III)(3-OMe-Sal_2_trien)][Au(dmit)_2_] [10]. The main difference between these two complexes is diverse valence of the metal ion in the M(dmit)_2−_^−^ anion, Ni(II) and Au(III), respectively, according to which the charge state of dmit is not the same: 1.5^−^ in **1** and 2^−^ in the complex with Au. This is testified by the difference in bond length values of sensitive to the charge double C=C bonds in dmit: The average C=C bond length is equal to 1.368(3) Å in [Ni(II)(dmit)_2_]^−^ and 1.348(3) Å in [Au(III)(dmit)_2_]ˉ. Therefore, [M(dmit)_2_]ˉ is a anion radical in the first case and an anion in the second case. For this reason, complex **1** should be a good candidate for conducting materials, unlike the isostructural dielectric complex with Au(III).

#### 2.1.2. [Fe(III)(3-OMe-Sal_2_trien)]NO_3_·H_2_O (**2**)

**Figure 6:**
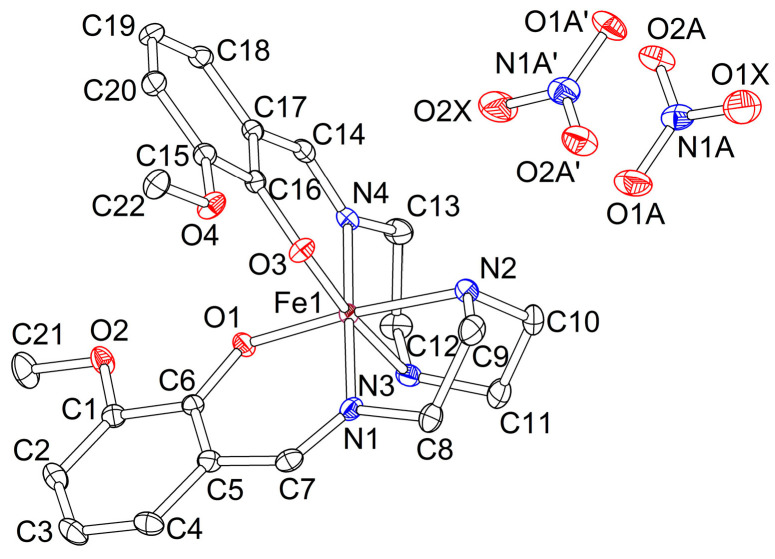
Asymmetric unit in **2** (120 K, ORTEP drawing with 50% probability ellipsoids). O1X and O2X atoms are mixed sites of water and NO_3_ molecules with 1:1 occupancy ratio. H-atoms are omitted for clarity.

Single crystals of the precursor compound **2** have monoclinic symmetry (space group *P*2_1_/*c*). The asymmetric unit contains one [Fe(3-OMe-Sal_2_trien)]^+^ cation, one NO_3_^−^ anion, and a water molecule, the last two being disordered in two equiprobable sites (Figure 6).

**Figure 7:**
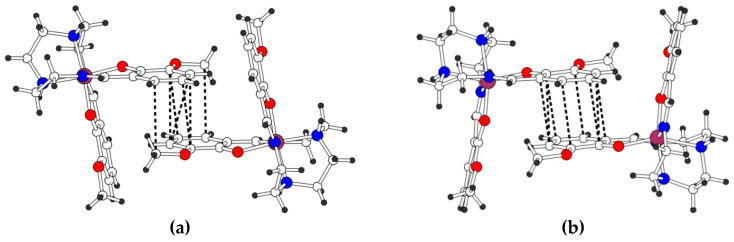
The *π*-*π* interacting cationic pair in **2**, 120 K (**a**) and **3**, 100 K (**b**). The C…C contacts < 3.6 Å are shown by dashed lines.

The X-ray crystal structure of **2** was determined at 293 and 120 K. The Fe(III) ion in [Fe(3-OMe-Sal_2_trien)]^+^ has a low-spin state at both temperatures. The geometry of the central FeN_4_O_2_ octahedron differs significantly from that in the HS complex **1** (Table 1). The Fe-N and Fe-O bonds are shorter in **2**, and the O-Fe-N angles are much closer to 180° [174.02(6) and 174.72(6)°]. The dihedral angle *α* between the salicylidene arms of the ligand is acute and equal to 75.84(4)°, and the central -CH_2_-CH_2_- bond (ii) of the trien is near parallel to the side bonds (i, iii) (Figure 5b), that are also prominent features of the LS complexes [10,25]. The cations are packed in the *π*-*π* interacting pairs, due to partial superposition of two parallel 3-OMe-salicylidene fragments. There are seven C…C contacts in the pair in the range 3.479(3)-3.587(3) Å (Figure 7a). The cations form several hydrogen contacts with surrounding H_2_O molecules and NO_3_ˉ anions of N-H…O (H…O 2.01–2.38 Å), C-H…O (H…O 2.36–2.61 Å), and C-H…N types (H…N 2.53 Å). All the numeric values are given for the 120 K structure, and they change a little at 293 K.

#### 2.1.3. [Fe(III)(3-OMe-Sal_2_trien)]I (**3**)

Single crystals of the precursor compound **3** have monoclinic symmetry (space group *P*2_1_/*n*). They are not isostructural to **2**. The asymmetric unit contains one [Fe(3-OMe-Sal_2_trien)]^+^ cation and one I^−^ anion, in general, positions. The atom numeration in the cation is exactly the same as in **2** (Figure 6).

The X-ray crystal structure of **3** was determined at 293 and 100 K. At both temperatures, the Fe(III) ion in [Fe(3-OMe-Sal_2_trien)]^+^ is expected to be in a high-spin state. The geometry of the central FeN_4_O_2_ octahedron is similar to that in complex **1**: Lengthened Fe-N and Fe-O bonds, stronger distortion of the O-Fe-N angles from 180° (Table 1), obtuse dihedral angle *α* and near-normal position of ii and i, iii bonds (Figure 5c). Like in the structure of **2**, the *π*-stacked pairs of the cations are observed, but with near-perfect superposition of aromatic rings with centroid…centroid distances of 3.551(2) Å and eight C…C contacts in the range 3.429(3)–3.578(3) Å at 120 K (Figure 7b). There are two cation…anion N-H…I contacts with H…I distances of 2.82 and 2.87 Å.

**Figure 8:**
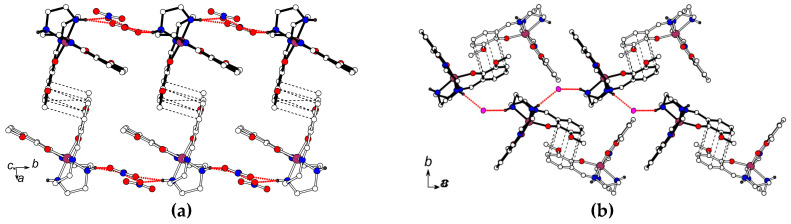
Cationic chains with N-H…anion hydrogen bonding (red dotted lines) in **2** (**a**) and **3** (**b**).

In both precursor compounds, the hydrogen bonding between two -NH functions of the trien moiety and anions results in a formation of infinite cationic chains that topology is found to be very different in **2** and **3** (Figure 8). The cations involved in the hydrogen-bonded chains (they are marked by black bonds) are packed in a side-to-side manner in the LS structure of **2** (Figure 8a) and in a head-to-head manner in the HS structure of **3** (Figure 8b). In both structures, one of the 3-OMe-salicylidene fragments of each cation is *π*-stacked to the same fragment of the nearest cation, while the second 3-OMe-salicylidene has *π*-stacking with the NO_3_^¯^ anions in **2** or with the cation in **3**. In the latter interaction, the interplane distance is large, 3.82(1) Å, and all the C…C distances are over 3.85 Å. Thus, in both **2** and **3**, one of two 3-OMe-salicylidene arms has stronger cooperation with the crystal environment than another one.

### 2.2. Magnetic Properties

#### Magnetic Susceptibility

**Figure 9:**
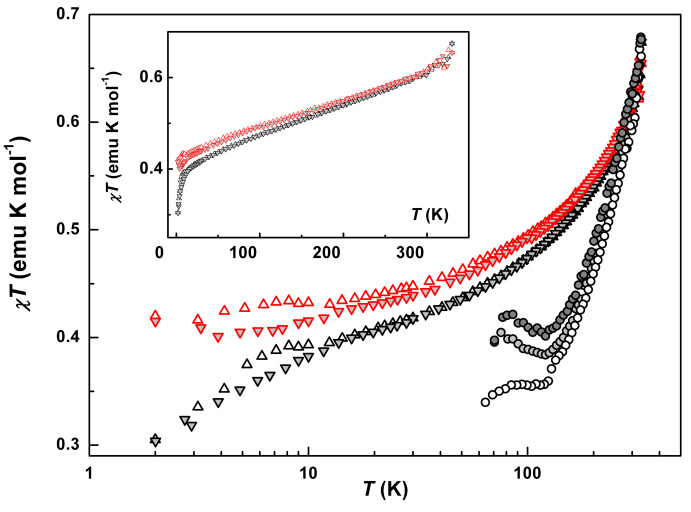
Temperature evolution of the product *χT* for **1** ((ο), (ο), (ο)—extracted values of (*χT)_LS_/γ_LS_* at different thermal cycles), **2** ((Δ)- warming, (∇)- cooling regimes) and **3** (∇, Δ) in logarithmic *T*-scale. Inset: plot *χT*(*T*) for **2** and **3** in linear *T*-scale.

The magnetic response of the precursors **2** and **3** was expected to result from the contribution of the Fe^3+^ cations, which can be either in low spin state *S* = 1/2 (LS) or high spin state *S* = 5/2 (HS). The temperature evolution of the *χT* products for both precursors is shown in Figure 9. At temperatures below 10 K *χT* ≈ 0.4 emu K/mol is close to the theoretical value of 0.375 emu K/mol corresponding to the paramagnetic response of magnetic moments *S* = 1/2 of LS Fe^3+^. A decline down to 0.3 emu K/mol at 2.0 K arose, due to antiferromagnetic interactions in **3**, whereas the local magnetic moments of Fe^3+^ ions in **2** remain non-interacting. This agrees with a different character of hydrogen bonding between two -NH functions of the trien moiety and anions in **2** and **3** (Figure 8). AFM interactions in **3** are consistent with the stronger linkage of the cations, due to uncompensated strong hydrogen bonding (N-H…I contacts with H…I distances of 2.82 and 2.87 Å) with adjacent iodine anion (100 K: *d*(NI) = 3.491 Å, *θ*(NHI) = 151.72°, bond energy by geometry factor: 2030) and *π*-stacking of neighboring 3-OMe-salicylidene fragments.

Gradual growth of *χT*, up to 0.68 emu K/mol at 350 K, is identical for both precursors despite their structural differences. Both curves are fully reversible in the range 10–350 K and have a weak hysteresis below 10 K. Such behavior was observed in similar structures and other polymorphic forms [5,18,19,28,29,30,31,32,33,34,35,36]. It was not associated with spin-crossover transition, unless the datapoints designate the exponential tail of a distant SCO transition. It also cannot be explained by the temperature drift of the *g*-factor [28]. Instead, this can be attributed to the contribution of HS Fe^3+^ ions with *S* = 5/2, possibly induced by the accumulation of structural distortions/defects in the H-bonded chains with the temperature. A discrepancy between the prediction of the ground spin state in **3** from structural data (HS) and the magnitude of the total spin response in *dc* SQUID (Superconducting Quantum Interference Device) measurements (LS) is significant and looks confusing. Crystallographic reasoning is based on formal linkage of the angle between the two 3-OMe-salicylidene arms of a “free” cation with the spin state of the Fe^3+^ ion. However, the configuration in **3** is distorted by counteraction of strong hydrogen bonding with iodine anion on the one hand, and *π*-stacking with the neighbor cation on the other one (Figure 8b). The overall distortions of the ligand field turned out to be insufficient to cause HS splitting of the *d*-states, in comparison with analogous effect in the non-substituted ligands [Fe(Saltrien)]^+^. On contrary to XRD predictions, the magnetic data give the factual gathering of the total spin response of the polycrystalline sample, which directly indicated LS type of the *d*-levels splitting. The value of angle α in the salts of Fe(III)sal_2_trien strongly depends on the counter ion and substitutes in the ligand. There are no systematic studies on the influence of the counter ion on the spin state (HS, LS) and α value in Fe(III) salts with 5-methoxy substituted sal_2_trien ligand. According to [37] the non substituted complexes [Fe(III)(sal_2_trien)]^+^ demonstrate LS state at 61.8° < α < 73.6°. The HS state was observed for 76.6° < α < 125.5°. So that, the compound [Fe(sal_2_trien)] ClO_4_ demonstrate HS state when α = 76.3°, while HS in [Fe(sal_2_trien)] BrF_4_ 0.5 C_2_H_4_Cl_4_ has α = 121.8°. In **3**, the angle α = 103.6° did not provide sufficient changes of the ligand field to induce HS spin configuration. We believe that this is a result of 5-methoxy substitution and strong H-bonding with iodine anion.

**Figure 10:**
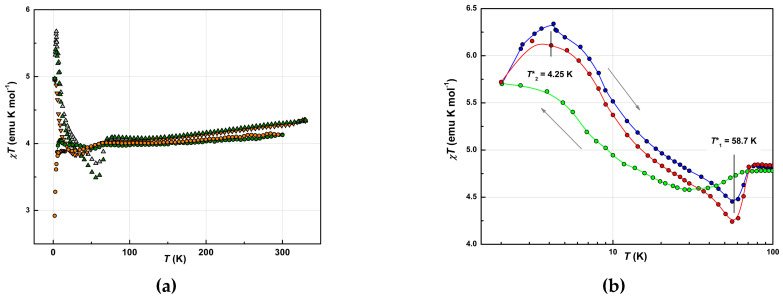
(**a**) Temperature dependences of the *χT* product for **1** in several sequential thermal cycles (Δ, Δ, ο)—warming, (∇, ο)—cooling regimes). (**b**) Temperature dependences of the product *χT_HS_/γ_HS_* for **1** (cycle 1: warming (ο) and cooling (ο); cycle 2: warming (ο)). The *χT_HS_* value at 100 K includes estimated contributions of the HS ferric moiety (82%) and formal contribution of the anion radicals Ni(dmit)_2_^−^. Logarithmic *T*-scale.

The magnetic properties of [Fe(III)(3-OMe-Sal_2_trien)][Ni(dmit)_2_] (**1**) were found strongly sensitive to the “thermal history”. Initial measurements were performed on the polycrstalline samples, while slow cooling (5–0.5 K/min depending on the *T*-range) from 293 K down to 2.0 K. At *T* = 2.0 K, the magnetization curves were recorded. Then, the magnetic susceptibility was measured again in a warming regime, *T* = 2.0–350 K. The measurements were repeated in several thermal cyclings (cooling-warming). Finally, the same samples were measured in several months. The measurements were also performed on batches from several syntheses, and on different SQUID machines. The temperature dependences of the *χT* product in the sequential measurement cycles are shown in Figure 10a.

Each curve is characterized by a reversible ascending segment at *T* ≥ 100 K, a plateau 60 K < *T* < 100 K, and an irreversible low-temperature segment at *T* < 60 K. The *χT* behavior above 60 K is distinctive for the SCO hybrids with Ni(dmit)_2_ alternating sub-lattices [23,28,29,30,31,32]. We have found that the plateau level varied depending on measurement cycles. It grew in several cooling-warming cycles and reached saturation at ~4.0 emu K/mol, including the aged samples. This irreversibility resembles the *χT* evolution in the isostractural SCO compounds with Au(dmit)_2_ and Au(dddt)_2_ counterions [10]. By analogy with the approach in that reference, we attributed the total magnetic response (*χT*)*_tot_* to a sum of contributions from LS fraction, *γ_LS_*(*χT*)*_LS_*, and HS fraction, *γ_HS_*(*χT*)*_HS_*, with the respective weight factors *γ_LS_* and *γ_HS_* = 1 − *γ_LS_*. Here, and further in the text, we would understand “fraction” in terms of partial volume of the crystal (multiply-connected domain) occupied by a certain spin-phase. By the “HS phase”, we would understand a type of the modified original structure, where all SCO complexes were irreversibly converted into a residual HS state, (*χT*)*_HS_*. Respectively, the “LS phase” corresponds to the virgin state, which is supposed to be LS, (*χT*)*_LS_*. Here we also assumed that according to a stoichiometry 1:1 a formal spin *S* = 1/2 belongs to the anion Ni(dmit)_2_^−^. The HS phase prevailed in the result of fractioning (Figure 10a), *γ_LS_*/*γ_HS_ =* 18/82, which is consistent with the crystallographic characterization of the individual single crystal (bulk HS). Note, that X-ray analysis did not reveal ~20% of LS fraction. This is explicable due to different thermal histories of the samples in X-ray and SQUID measurements. To a certain extent, the spin-phases do not represent crystallographically different structures. We presume the fractioning process was also taking place during sample transportation between the labs. So that, the dry polycrystalline powders delivered for SQUID and EPR measurements already contained a mixture of LS and HS phases. Though a variable ratio of co-existing LH and HS phases was present in the same sample, we did not observe nor a partial SCO transition, nor its pronounced segment. We believe that fractioning, as intrinsic inhomogeneity, replaced SCO.

A gradual slope above 100 K on the *χT* curve was attributed to the LS phase similar, to the one in the precursors. Thermo cycling irreversibly reduced this fraction. Indeed, subtraction of the paramagnetic HS fraction and restoring LS contribution to 100% resulted in the behavior similar to the one in the bulk of both precursors (Figure 9, *S* = 1/2 contribution from Ni(dmit)_2_ was also subtracted). Of course, Ni(dmit)_2_ network differentiates the hybrid structure from the precursors, so the plateau extended to higher temperatures, *T* ≈ 60 K. Meantime, a character and magnitude of the gradual *χT* growth remained reversible and unchanged during *T*-cyclings. Spin-spin interactions between both counterions spin reservoirs are insignificant, where Fe^3+^ exists in the LS state (further EPR experiments proved this, as the respective signal exhibited Curie type behavior of the intensity, *I*_EPR_*T* = const, Figure S1).

A significant increase in the *χT* value at helium temperatures and a decline below 60 K were attributed to the fraction of the HS phase. Indeed, the magnitude of these changes exceeds the value of the total paramagnetic response of *S* = 1/2. Therefore, we disengaged LS contribution and further analyzed the low-temperature segment in the scale as if the HS phase occupies 100% volume, (*χT)_HS_/γ_HS_*. The normalized data from sequential thermocycles, shown in Figure 10b, allowed quantitative analysis. The evolution of (*χT)_HS_* contributions proves that the magnetic ground state of the HS fraction developed upon a thermal history of the sample. Similar behavior was observed on hybrid SCO systems with Au(dmit)_2_ and Au(dddt)_2_ [10]. Two pronounced curves were obtained at the ratio *γ_LS_/γ_HS_* = 18/82. At maximum, *T*_max_ = 4.25 K, the estimated *(χT*)*_HS_* value reached 6.4 emu K/mol, which is close to 6.0 emu K/mol, corresponding to ferromagnetically coupled spins *S* = 3. At a minimum, *T_min_* = 58.7 K, *(χT*)*_HS_* dropped below the plateau on a value Δ(*χT*)*_HS_* ≈ 0.5 emu K/mol, which is close to the paramagnetic contribution of *S* = 1/2 spins. This contribution might be related to the Ni(dmit)_2_ radicals. Then, the drop off is reasonable to link with the result of superexchange interactions between Ni(dmit)_2_ and HS [Fe(III)(3-OMe-Sal_2_trien)] spin sub-systems. Respectively, FM coupled spin pairs *S* = 1/2 and 5/2 would act as effective spins *S_eff_* = 3 at helium temperatures, giving rise to a new superparamagnetic curve (*χT)_HS_*. Note that the anisotropy of the effective magnetic moment would also change compare to the initial single-ion ones.

### 2.3. Magnetization Curves

**Figure 11:**
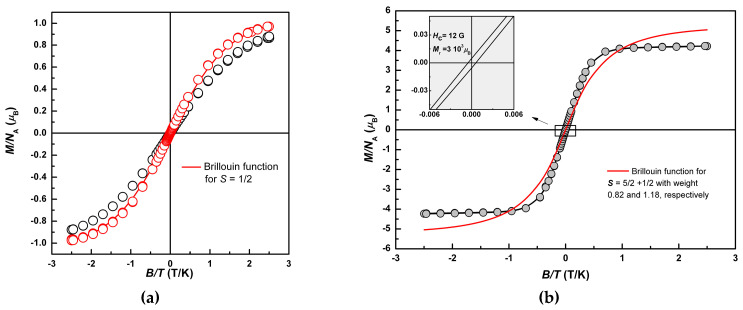
Field dependence of the magnetization, *M*(*B/T*), for **2** (○), **3** (○) (**a**) (solid line is a Brillouin function for *S* = 1/2), and for **1** (**b**) (solid red line is the sum of Brillouin functions corresponding to a weighted superposition of for *S* = 1/2 (18%) and *S* = 5/2 (82%)) at *T* = 2.0 K.

The magnetization curves, *M* = *M_z_*(*B/T*), measured at *T* = 2.0 K by changing the magnitude of the magnetic field in the range *B* = −5.0–5.0 T, are presented in Figure 11a (**2**,**3**) and Figure 11b (**1**). Magnetic field dependences for **2** and **3** are consistent with respective temperature dependences of the magnetic susceptibility, in terms of confirmed LS ground state for both precursors. The data points for **2** were well described by the Brillouin function with *S* = 1/2 (solid line on Figure 11a). The data points for **3** lay noticeably below the Brillouin curve with lower slope at small field values −1.5 T < *B* < +1.5 T. This agrees with suggested AFM interactions as a cause of the *χT* decline for **3**.

The shape of the *M*(*B/T*) curve for **1** varied depending on the thermal history, in accordance with the low temperature changes of (*χT*)*_HS_* in Figure 10b. The most identifiable curve was obtained after several warming-heating cycles, at which (*χT*)*_HS_* demonstrated a maximum of 6.4 emu K/mol. It exhibited sharper slopes (compare to the Brillouin curves), hysteresis with the coercive field, *H_c_* = 12 G, remnant magnetization *M*_r_ = 3 × 10^−3^ *μ*_B_, and pronounced saturation at the level *M_s_* = 4.1*μ*_B_. Field sweep rate in the range 1.5 T < *B* < +1.5 T was 200 G/min and 5000 G/min at |*B*| ≥ 1.5 T. Hysteresis and saturation at *M_s_* ≈ 2/3·6*μ*_B_ validate the FM coupling in spin pairs *S* = 1/2 and 5/2, and formation of the effective spin moments *S_eff_* = 3 at helium temperatures This also confirms considerable anisotropy of these moments. The magnetization curves for intermediate *T*-cycles are presented in Figure S2.

### 2.4. Transport Properties

Electrical resistance in the single crystals of **1** was measured in two configurations: “Transverse”, when the current *J* was directed along the *c*-axis, and “in-plane”, when the current was set along the (*ab*) plane of Ni(dmit)_2_ layers (see Figure 3). Two types of single crystals were used for the measurements: The ones elongated in *c*-direction, and the other ones—elongated in (*ab*) plane. The resistivity along the *c*-axis, *ρ*_||c_ (293 K), reached the value of 2 × 10^5^ Ohm·cm, whereas the in-plane value, *ρ*_⊥c_ (293 K), was much lower, 7 × 10^2^ Ohm·cm. The anion radical layers in single fresh crystals of the 1:1 stoichiometric hybrid compound happened to be not insulating, but remarkably conducting. The value of quasi-two-dimensional anisotropy of the resistivity at 293 K reached ~3 × 10^2^.

**Figure 12:**
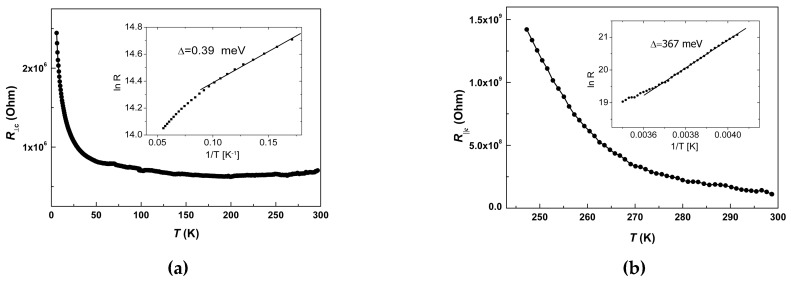
The temperature dependences of the “transverse” (**a**) and “in-plane” (**b**) resistance, *R*_||c_(*T*) and *R*_⊥c_(*T*). Insets: Extraction of energy barrier values, Δ_⊥c_ = 0.39 meV and Δ_||c_ = 367 meV, by Arrhenius law simulations.

The temperature dependences of the “transverse” and “in-plane” resistance, *R*_||c_(*T*) and *R*_⊥c_(*T*), are presented in Figure 12. Both dependences had extended segments complying with the Arrhenius law. However, the values of activation energy, Δ were essentially different: Δ_⊥c_ = 0.39 meV, Δ_||c_ = 367 meV. Note, that the latter value is ~1/3 eV, whereas the typical HOMO-LUMO gap value of Ni(dmit)_2_ sublattice is of the order of ~1 eV. On the contrary, the in-plane value of Δ_⊥c_ of ~0.4 meV fits in the ballpark of a variety of conducting Ni(dmit)_2_ salts with fractional oxidation [8,9]. Both “deep” and “shallow” activation transport does not seem realistic without mediating the role of the metallocomplex cations. As a result of charge transfer from the donor [Fe(III)(3-OMe-Sal_2_trien)] complex, the unpaired electron occupies the near-edge molecular orbitals of the LUMO band of Ni(dmit)_2_ network. Thermoactivated charge disproportionation would require much less energy, compared to electron hopping between anion radicals. This mechanism may facilitate not only the in-plane electron transport, but also the transverse one, though at higher energy barriers. Raman spectroscopy of the single crystals **1** at changing temperatures might be helpful in further clarification of the transport mechanism.

**Figure 13:**
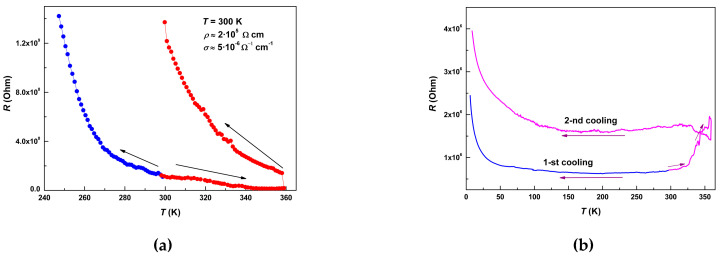
Evolution of the temperature dependences of the “transverse” (**a**) and “in-plane” (**b**) resistances, *R*_||c_(*T*) and *R*_⊥c_(*T*), during first cycles of warming and cooling.

**Figure 14:**
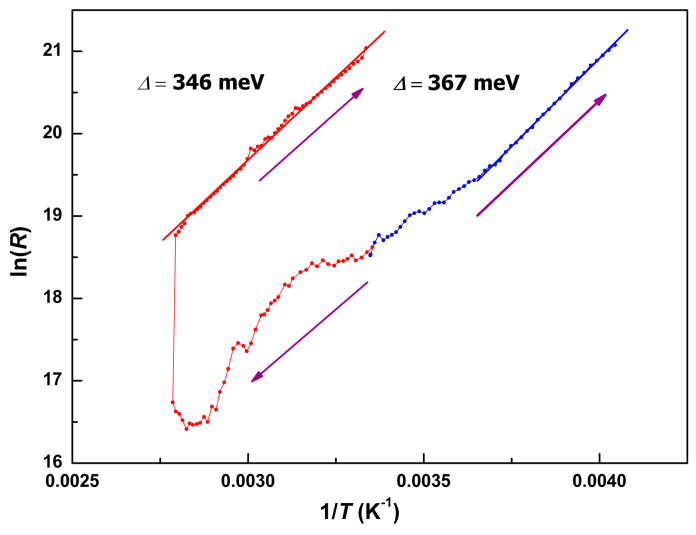
Extraction of energy barrier values, Δ_||c_ = 367 and 347 meV, by Arrhenius law simulations after several warming-cooling cycles.

Repeated measurements, accompanied by sequential warming-cooling cycles in the range 4–360 K, led irreversibly to the drastic growth of the resistance values, both *R*_||c_(*T*) (Figure 13a) and *R*_⊥c_(*T*) (Figure 13b). In the second loop, the ‘transverse” resistivity, *R*_||c_, remained detectable only above 300 K, reaching a minimal value of 2 × 10^8^ Ohm at 360 K. The “in-plane” resistivity, *R*_⊥c_, nearly doubled the value of its residual level, *R*_RES_. Qualitatively this behavior resembles conducting properties of the composite material consisting of the conducting balls in a dielectric matrix, at changing (reducing) number of the balls. Trivial scheme of parallel and serial connections of resistances in electrical engineering qualitatively explains the observed evolution: *R_tot_* = *R*_res_ + *R*_1_*R*_2_ / (*R*_1_ + *R*_2_), where *R*_1_ and *R*_2_—resistances in parallel connection, *R*_res_—resistance in serial connection. According to the magnetic data, thermocycling changes the topology of fractions and the volume ratio, *γ_LS_*/*γ_HS_*, towards a sustained balance at ~80% of the HS phase. Hence, fresh crystal had dominant LS phase, demonstrating quasi-two-dimensional conductivity in (*ab*) plane of Ni(dmit)_2_ layers, *R*_||c,__⊥c_(*T*)~*R*_0||,__⊥_·exp(Δ_||c,__⊥c_/*k*_B_*T*) (*k*_B_—Boltzman constant). Upon thermo-induced phase segregation fraction of the dielectric HS phase increases until percolation takes place. Then, the increasing *R*_res_ component rules the *dc* transport properties, until the crystal becomes an insulator. This simplified model satisfactory describes the evolution of the observed transport properties and deviation from the Arrhenius law. This may also explain the resistivity curve in [29,30,31,35,38]. Note that the value of the energy barrier should not change critically. Indeed, Δ_||c_ changed insignificantly from 367 meV to 347 meV during the first full cycle, shown in Figure 14. This analysis strongly supports reasoning of the magnetic section. The polycrystalline powder analyzed in magnetic experiments, *γ_LS_*/*γ_HS_* ≈ 1/4, consisted of insulating crystals with conducting inclusions in bulk. EPR, as a locally sensitive probe, would be a useful tool for further clarification.

### 2.5. EPR Properties

**Figure 15:**
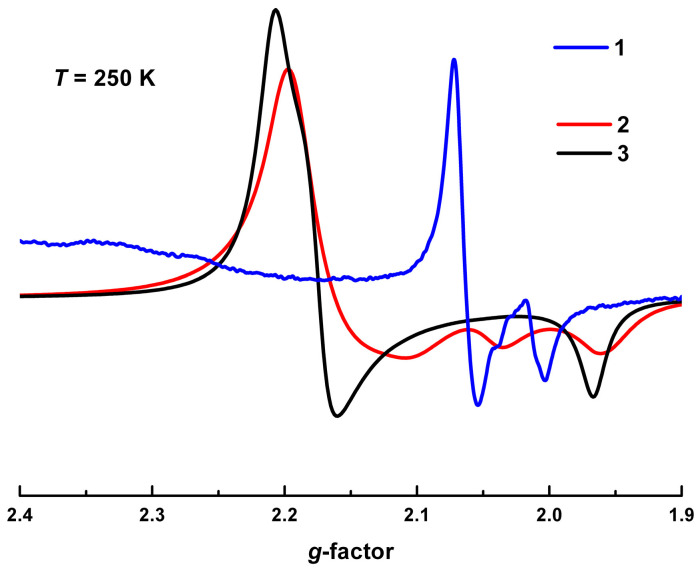
Comparison of the EPR spectra for **1**, **2**, and **3** in the vicinity of *g* ~2.0 at 250 K.

X-band EPR spectra of the precursors **2** and **3** are presented in Figure 15. For comparison, a part of the spectrum of **1** in the range *g* = 1.9–2.2 is also shown on the same scale. At 250 K the spectra of the precursor polycrystals consist of a single line with anisotropic *g*-factor *g_1_^LS^* = 2.164, *g_2_^LS^* = 2.036, *g_3_^LS^* = 1.961 for **2**, and *g_1_^LS^* = 2.207, *g_2_^LS^* = 2.175, *g_3_^LS^* = 1.966 for **3**. They are fairly consistent with *g*-values corresponding to LS spin state of Fe^3+^ ions in simple salts with sal_2_-trien type SCO cations and similar ligands [28,39,40].

The line shapes indicate that the ligand field in **3** had a different character of symmetry in comparison with **2**. However, the total anisotropy magnitude (i.e.*, t*_2g_–*e*_g_ splitting magnitude) is the same, Δ*g^LS^* ≈ 0.24. Hence, EPR spectra not only confirm the LS state for **3**, but also support the assumption about remaining significant splitting of the *d*-levels in the ligand field, high enough to maintain the LS state. This is true despite the wide angle between 3-OMe-salicylidene fragments, usually attributed to the HS state. The temperature evolution of these spectra and their respective g-values were presented in Figures S3 and S4. The *g*-values did not change significantly with the temperature, as well as the linewidth, Δ*B_2_^LS^*(100 K) = 120 G (**2**), 32 G (**3**), where Δ*B_2_* corresponds to the linewidth of the *g_2_^LS^* component (Figures S5 and S6). The relative double integrated intensity of the total spectrum followed Curie law down to nitrogen temperatures. Contributions from Fe^3+^ ions in the HS state were not detected, which agrees with their very low concentrations (< 6%, according to the analysis of *χT*).

The total spectrum of **1** consists of more than 10 lines. Low- and high-field segments of the spectrum and respective simulations are shown in Figure S7. A variety of lines in a broad magnetic field range, *B* = 0–13 kG, reflects a complex energy levels diagram with multiple spin-transitions. Their detailed interpretation is beyond the scope of current work. Qualitatively this indicates the presence of several interacting spin ensembles [41,42]. This strongly supports our assumption about fractioning and spin-spin interactions in the HS phase.

**Figure 16:**
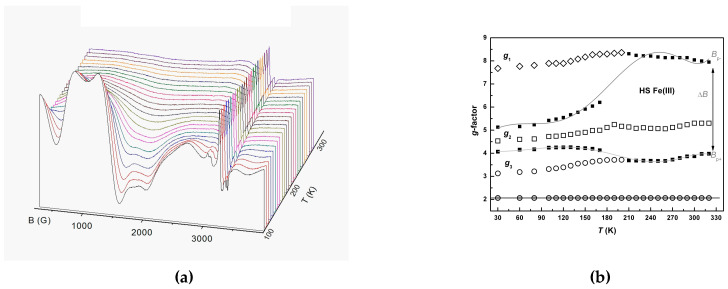
(**a**) Temperature evolution of the EPR spectrum (lower field part) for **1**. (**b**) Temperature evolution of the *g*-factors associated with the major spectral lines from the ferric moiety of **1**: (◊)-*g*_1_, (□)-*g*_2_, (○)-*g*_3_ belong to the contribution of the HS fraction; (●)—*g* ~ 2.0 belongs to the dominant line in the spectrum of the LS fraction. Solid lines connecting squares (■) designate positions of the peaks *B*_p+_ and *B*_p−_ for a simulated central line of the spectrum, corresponding to *g*_2_. The peak-to-peak linewidth of the central line is Δ*B* = *B*_p+_ − *B*_p−_.

The signal corresponding to the *S* = 1/2 moments of the LS Fe^3+^ fraction was consistent with those from the precursors, *g*_⊥_*^LS^* = 2.06, *g*_||_*^LS^* = 2.01 (Figure 15). It had an axial anisotropy shape, though with weaker *g*-splitting, Δ*g^LS^* = 0.05. The characteristics of this signal did not change substantially with the temperature, as well as the ones of the precursors. This is seen from the waterfall presentation of spectral evolution in Figure 16a.

The narrow signal (LS) sits in the base of a broader set of lines centered around *g* ~ 2.0. Taking into account that the LS phase is semiconducting, we did not expect observation of *S* = 1/2 moments of Ni(dmit)_2_ anion radicals. X-band EPR spectra of regular Ni(dmit)_2_ structures in simple salts were very rarely observable, unless those arousing from defects [8,9,39]. Meanwhile, we also observed the narrow EPR line coming from defects in Ni(dmit)_2_ sublattice. It was reliably identified by a specific hyperfine doublet associated with the sulfur atoms of (dmit)_2_ ligand (Figure S7). Its intensity was negligible compare to the other lines, and we omitted its discussion in the current work. Thus, the broader set of lines does not belong to the LS phase.

Spatial distribution of the LS/HS fractions could not be reconstructed from SQUID measurements. However, it was reasonably suggested that mutual topology changed during thermal cyclings. This came not only from the cooperative effect among cations, but also from cooperativity with the metal-dithiolate network, as was shown by the evolution of transport properties. If so, the properties of the spin moments of Ni(dmit)_2_ in the HS phase had to be different from those in the LS phase. Then, some anion radicals occupying the “boundaries” between phases would become “defects” in terms of magnetic moments on Ni(dmit)_2_. Evidently, we observed these “defects” as a narrow, weak line with the hyperfine structure of ^33^S satellites (Figure S8) [39]. As we deducted from the transport measurements, the HS phase is insulating. Hence, spin moments of Ni(dmit)_2_ are localized. Moreover, from the low temperature part of the *χT*(*T*) curve, we assumed the exchange interactions between Ni(dmit)_2_ and HS Fe^3+^ spin systems. Such interactions would certainly give rise to multiple spin transitions, especially considering considerable anisotropy of *S* = 5/2 spins. Indeed, EPR signal of *S* = 5/2 moments associated with HS Fe^3+^ ions revealed anisotropic structure *g_1_^HS^* = 8.207, *g_2_^HS^* = 5.079, *g_3_^HS^* = 3.647. Temperature evolution is presented in Figure 16b. Here the black points in the figure depict positions on the slopes of the simulated central component (*g_2_^HS^*). By using these data, we estimated the partial linewidth value. The evolution of the extracted linewidth, Δ*B_2_^HS^*(*T*), is shown in Figure S9. By also considering the anisotropy, the interactions with *S* = 1/2 local moments, and polycrystalline form of the sample, the multiline total EPR spectrum was qualitatively understood.

Set of broader lines centered at *g* ~ 2.0 demonstrated a significant evolution of the shape in the course of thermocycling. A comparison of several spectra is shown in Figure S8. We have reason to believe that its structure was the most pronounced when *χT*(*T*) demonstrated the FM peak at 4.25 K. A determination of precise experimental correspondence was complicated, due to irreversible changes in the properties.

## 3. Materials and Methods

### 3.1. General

Commercial solvents were used without further purification unless otherwise specified. 3-methoxysalicylaldehyde (o-vanillin), 1,8-diamino-3,6,-diazaoctane (trien-oil), Fe(NO_3_)_3_·9H_2_O, NaOCH_3_ were purchased from commercial sources and used without further purification. Complexes [(Bu)_4_N][Ni(dmit)_2_] were prepared according to the literature [43,44].

### 3.2. [Fe(3-OMe-Sal_2_trien)][Ni(dmit)_2_] (1)

A solution of [Fe(3-OMe-Sal_2_trien)]NO_3_·H_2_O (53.00 mg, 0.097 mmol) in acetonitrile (15 mL) was added dropwise to the stirred solution of [(n-Bu)_4_N][Ni(dmit)_2_] (69.00 mg, 0.1 mmol) in acetonitrile (15 mL). After being placed for one week at ambient temperature, the resulting precipitate was gravity filtered and dried in vacuo. The compound **1** (Yield 72.47 mg, 81.3%) was obtained in the form of black plate-like crystals, suitable for X-ray diffraction (Figure S10). Anal. Found: C, 36.48; H, 3.11; N, 6.18%. Calcd for C_28_H_28_FeN_4_NiO_4_S_10_: C, 36.56; H, 3.05; N, 6.09%. The RSMA information on the elements’ proportion is Fe:Ni:S = 1:1:10.

### 3.3. [Fe(3-OMe-Sal_2_trien)]NO_3_·H_2_O (2)

Complex **2** was synthesized by following the previously published procedure [44]. By a template synthesis, through the reaction of 1,8-diamino-3,6,-diazaoctane (trien-oil) with 2 equiv. of 3-methoxysalicylaldehyde and 1 equiv. of Fe(NO_3_)_3_·9H_2_O in MeOH. A solution of 1,8-diamino-3,6,-diazaoctane (trien-oil, 10 mmol) dissolved in methanol (10 mL) was added to a solution of 3-methoxysalicylaldehyde (20 mmol) in methanol (40 mL) and the resulting yellow mixture stirred for 10 min. Sodium methoxide (NaOCH_3_) (20 mmol) in methanol (50 mL) was slowly added. Iron nitrate nonahydrate Fe(NO_3_)_3_·9H_2_O (10 mmol) in methanol (25 mL) was added dropwise to the stirred solution. The resulting a dark purple solution was gravity filtered, solvent removed under reduced pressure to give a purple solid. The crude solid was recrystallized from warm water afforded shiny black microcrystals, suitable for X-ray diffraction, which were collected and dried in vacuo over P_2_O_5_.Yeld 37.5%. Anal. Found: C, 47.73; H, 5.25; N, 12.74; Fe, 10.43%. Calcd for C_22_H_28_FeN_5_O_7_·H_2_O: C, 48.18; H, 5.47; N, 12.77; Fe, 10.21%.

### 3.4. [Fe(3-OMe-Sal_2_trien)]I (**3**)

Preparation of [Fe(3-OMe-Sal_2_trien)]I was prepared by reaction of metathesis from NO_3_^−^ (**2**) salt. To 1 mmol of [Fe(3-OMe-Sal_2_trien)]NO_3_·H_2_O (**2**) salt dissolved in 30 mL of warm water was added 30 mL of an aqueous solution containing 40 mmol KI at room temperature. Black crystalline product was obtained by cooling the solution at 0 °C for ~3 h. The raw fraction was recrystallized from 150 mL of warm water, suitable for X-ray diffraction shiny black crystals collected by filtration, washed with an acetone-ether mixture (1:3 by volume) 50 mL, dried under vacuum at room temperature over P_2_O_5_ for 16 h. Yeld 20.5%. Anal. Calcd for C_22_H_28_FeN_4_O_4_I: C, 44.35; H, 4.70; N, 9.41; O, 10.75; Fe, 9.41%. Found: C, 43.79; H, 4.90; N, 9.36%; Fe, 9.23%. The *RSMA* information on the elements’ proportion is Fe:I = 1:1.

### 3.5. Electron-Probe X-ray Spectral Microanalysis (RSMA)

The preliminary compositions of single crystals of **1**,**3** were determined by the RSMA method on a JEOL JSM-5800L scanning electron microscope (JEOL Ltd., Tokyo, Japan) with a thousand-fold magnification and electron beam energy of 20 keV. The depth of beam penetration into the sample was 1–3 µm. *RSMA* provides direct information on the elements Fe, Ni, S, I present.

X-ray powder diffraction pattern (*P-XRD*) of **1**,**2** was collected on the Siemens D500 powder diffractometer (Siemens AG, Karlsruhe, Germany) with Bragg-Brentano geometry using Cu Ka1 radiation (λ = 1.5418 Å). Powder patterns were used as a fingerprint to identify the crystalline phases present in the polycrystalline material (Figures S11 and S12). The diffractogram of **3** (Figure S13) was recorded on a Bruker D8 Advance Vario diffractometer (Bruker AXS GmbH, Karlshruhe, Germany) equipped with an X-ray tube with a copper anode and a Ge (111) monochromator (CuK_1) and a LynxEye position-sensitive detector, in transmission installations. The shooting interval was 4–60°, step 0.02°. The analysis was performed using the Bruker Topas 5 [45].

### 3.6. Optical Characterization

Microphotographs of the ground crystals are shown in Figure S14a–c for **1** (violet color), **2** (green color), **3** (green color), respectively. Black shiny single crystals of **3** (Figure S15a) were collected for X-ray diffraction. The polycrystalline powder containing single crystals and fragments of irregular shape (Figure S15b) was selected for SQUID and EPR measurements, including subsequent P-XRD. The sample image of the crystal taken for RSMA is presented in Figure S15c.

### 3.7. X-ray Crystallography

X-ray single crystal diffraction studies were carried out on a Bruker SMART APEX2 CCD diffractometer (Bruker AXS Advanced X-ray Solutions GmbH, Karlsruhe, Germany) for **1**,**2** at 120 K and **3** at 100 K and an Oxford Diffraction Gemini-R diffractometer ((Oxford Diffraction, Oxford, Oxfordshire, United Kingdom) with Atlas CCD detector for **2** and **3** at 293 K [λ(MoKα) = 0.71073 Å, graphite monochromator, ω-scan mode]. The structures were solved by the direct method and refined by the full-matrix least-squares technique against *F*^2^ in the anisotropic approximation for all non-hydrogen atoms using SHELX-2016 program packages [46]. Hydrogen atoms were localized from the Fourier synthesis of the electron density and refined in the isotropic approximation. Table 2 contains unit cell parameters and details of data collection and structure refinement. CCDC 2031156–2031160 contains supplementary crystallographic data for this paper. These data can be obtained free of charge from the Cambridge Crystallographic Data Centre, 12 Union Road, Cambridge CB21EZ, U.K. (fax (+44)1223-336-033; E-mail deposit@ccdc.cam.ac.uk), via www.ccdc.cam.ac.uk/data_request/cif.

### 3.8. Thermogravimetric Analysis

The thermogravimetric analysis was performed in the argon atmosphere with a heating rate of 5.0 °C min^−1^ using a NETZSCH STA 409 C Luxx thermal analyzer (NETZSCH-Geratebau GmbH, Selb, Germany), interfaced with a QMS 403 Aelos mass spectrometer (NETZSCH-Geratebau GmbH, Selb, Germany), which allows simultaneous thermogravimetry (TG), differential scanning calorimetry (DSC), and mass-spectrometry measurements.

The thermogram of [Fe(3-OMe-Sal_2_trien)](NO_3_)·H_2_O [2 H_2_O] is shown in Figure S16a. It was found that with increasing temperature, the loss of water molecules takes place in two steps. The first water weight loss of 3.91% was observed in the temperature range 50–170 °C with DSC endothermic peak at 158 °C. The second step (1.35%) occurs between 170–200 °C with a DSC exothermic peak at 180.7 °C (Figure S16a). As this takes place, the ions with *m*/*z* 18 (H_2_O) and 17 (HO) relating to the fragments of water molecules are observed in the gas phase in the mass spectrum. On heating above 200 °C, the complex begins to decompose.

The thermogram of [Fe(3-OMe-Sal_2_trien)]I (**3**) demonstrates a mass loss of 19.18% in the temperature range 50–400 °C with two DSC-peaks: Endothermic peak at 183.3 °C and exothermic peak at 271.1 °C, which is assigned to the decomposition of **3** (Figure S16b).

The thermogram of [Fe(3-OMe-Sal_2_trien)][Ni(dmit)_2_] (**1**) demonstrates a mass loss of 20.73% in the temperature range 50–270 °C with two *DSC*-peaks: Exothermic peak at 236.1 °C and endothermic peak at 258.1 °C, which is assigned to the melting of **1** (Figure S16c). The decomposition of **1** starts above 260 °C (*DSC* exothermic peak at 289.3 °C) and in the mass spectrum recorded in the gas phase, are observed the peaks relating the fragments of the complex molecule (CH: *m*/*z* = 13; CN-fragments: *m*/*z* = 26).

### 3.9. Measurements of Magnetic Properties

Variable-temperature magnetic susceptibility measurements were performed by using a Quantum Design MPMS-5-XL SQUID magnetometer (Quantum Design, San Diego, CA, USA). The static magnetic susceptibility *χ*(*T*) of the polycrystalline sample was measured at the magnetic fields *B* = 0.1 T, 4.0 T at warming and cooling regimes in the temperature range of 2–400 K. Field dependence of the magnetization *M*(*B*) were obtained at 2.0 K after several scans over the field range from −5.0 to +5.0 T. The sample had been cooled to 2.0 K in a magnetic field *B* = 4.0 T. Then, the measurements were performed at decreasing field with a sign reversal to −5.0 T and further increasing field to +5.0 T.

### 3.10. Measurements of Electrical Properties

The *dc* resistivity measurements were performed on single crystals by a four-probe method. Four annealed platinum wires (0.02 mm in diameter) were attached to a crystal surface by a graphite paste with two pairs of contacts attached to the opposite sample surfaces. We have used this contact geometry, because the crystals were too small (their size did not exceed 0.3 mm) for the standard 4-probe method, when four contacts are glued on one sample surface. The samples were placed in a cryostat with the variable temperature insert. Monitoring of the temperature variations and data acquisition was achieved by using a LakeShore temperature controller (Lake Shore cryotronics, Westerville, OH, USA) and an Agilent multimeter (Agilent, Santa Clara, CA, USA), driven by a PC.

The electrical resistance of single crystals was measured in the conducting plane using a four-probe technique on an automated setup with *dc* in a temperature range of 300–5 K for **1**. Platinum wire contact (*d* = 10µm) were glued to the crystal using the DOTITE XC-12 graphite paste.

### 3.11. Measurements of EPR Properties

EPR spectra were recorded in the temperature range of 90–370 K on a standard homodyne *X*-band (9.4 GHz) Bruker ELEXSYS E580 FT/CW spectrometer (Bruker AXS GmbH, Karlshruhe, Germany). The temperature was set and stabilized at a rate of 0.5–5 K/min with an accuracy of 0.1 K using a liquid nitrogen gas-flow cryostat (Bruker AXS GmbH, Karlshruhe, Germany). The spin contribution to the magnetic susceptibility was determined by the double integration of the EPR signal (Schumacher-Slichter method) under conditions for the field sweep Δ*B*_sw_ ≥ 10Δ*B* (Δ*B* is the peak-to-peak EPR line width of the total spectrum). In this case, an error of the method for the Lorentz line shape is ~10%. The pyrolytic coal product with *g* = 2.00283 was used as the standard of a spin concentration.

## 4. Conclusions

In this study, three compounds based on [Fe(3-OMe-Sal_2_trien)]^+^ SCO units were obtained and structurally characterized: [Fe(3-OMe-Sal_2_trien)][Ni(dmit)_2_] (**1**), [Fe(3-OMe-Sal_2_trien)]NO_3_·H_2_O (**2**), [Fe(3-OMe-Sal_2_trien)]I (**3**). All three did not exhibit a resolved homogeneous SCO transition. The hybrid structure with Ni(dmit)_2_ anion radical sublattice (**1**) exhibited spin-phase segregation (fractioning to LS and HS phases) and anisotropic electric conductivity. The two precursors of the hybrid structure, simple salts with nitrate and iodine anions (**2**,**3**), exhibited LS ground state. In particular, the structure of **3** revealed an unusually wide angle between the phenyl arms, α = 103.6 ° at room temperature. This was explained by strong H-bonded chains with Iodine anions and 5-Methoxy substitution in the ligand.

Despite 1:1 stoichiometry, the salt **1** was conducted with the anisotropic resistivities *ρ*_||c_ (293 K) = 2 × 10^5^ Ohm·cm and *ρ*_⊥__c_ (293 K) = 7 × 10^2^ Ohm·cm, *ρ*_||c_/*ρ*_⊥__c_ = 3 × 10^2^. Temperature dependences, *R*(*T*), in orthogonal directions, contained the fragments complying with the Arrhenius law. Quasi-two-dimensional character of the anisotropy also revealed itself in the values of activation energy: Δ_⊥__c_ = 0.39 meV, Δ_||c_ = 0.37 eV. Remarkably, the evolution of *R*(*T*) in a single crystal occurred highly irreversibly, depending on its thermal history. In accordance with the results of magnetic and EPR measurements on polycrystals, fractioning was observed in the transport properties of the individual single crystal.

Evolution of the partial contribution of the HS phase to the spin susceptibility of **1**, (*χT*)*_HS_*, revealed superexchange interactions between spins of the anion radicals, *S* = 1/2, and local magnetic moments of the Fe^3+^ ions, *S* = 5/2. The pronounced peak of (*χT*)*_HS_*/*γ_HS_* = 6.4 emu K/mol spoke in favor of super paramagnetism of the effective spin moments *S_eff_* = 3 at *T* < 4.25 K, suggesting a ferromagnetic coupling between the spin systems. Magnetization curves exhibited sharp slopes (compare to the Brillouin curves), hysteresis, and pronounce saturation at the level *Ms* = 4.1 ≈ 2/3 × 6*μ*_B_.

## Figures and Tables

**Table 1:** Selected bond lengths (Å) and angles (°) in [Fe(III)(3-OMe-Sal_2_trien)]^+^.

	1, 120 K	2, 120 K	2, 293 K	3, 100 K	3, 293 K
Fe1 O1	1.909(2)	1.873(1)	1.874(1)	1.917(1)	1.915(2)
Fe1 O3	1.913(2)	1.873(1)	1.873(1)	1.915(1)	1.905(2)
Fe1 N1	2.120(2)	1.926(1)	1.927(1)	2.119(2)	2.111(3)
Fe1 N2	2.173(2)	2.005(2)	2.006(1)	2.167(2)	2.184(3)
Fe1 N3	2.171(2)	1.996(1)	2.001(1)	2.175(2)	2.171(3)
Fe1 N4	2.103(2)	1.924(1)	1.927(1)	2.117(2)	2.109(3)
O1 Fe1 O3	105.41(8)	95.61(6)	95.48(4)	106.21(6)	105.4(1)
O1 Fe1 N1	86.45(8)	94.30(6)	94.08(4)	84.93(7)	85.0(1)
O1 Fe1 N2	160.47(8)	174.02(6)	173.90(5)	160.73(7)	160.8(1)
O1 Fe1 N3	93.70(8)	89.23(6)	89.34(5)	94.74(7)	95.0(1)
O1 Fe1 N4	88.92(8)	86.32(6)	86.63(4)	86.37(7)	86.7(1)
O3 Fe1 N1	96.43(7)	87.02(6)	87.23(4)	100.56(6)	100.1(1)
O3 Fe1 N2	87.96(8)	90.07(6)	90.23(5)	86.53(7)	86.9(1)
O3 Fe1 N3	155.23(8)	174.72(6)	174.62(5)	152.92(7)	153.6(1)
O3 Fe1 N4	87.34(8)	93.24(6)	93.16(4)	87.41(7)	87.6(1)
N1 Fe1 N2	77.81(8)	84.12(7)	84.04(5)	78.43(7)	78.3(1)
N1 Fe1 N3	100.42(8)	94.74(6)	94.85(5)	98.16(7)	98.2(1)
N1 Fe1 N4	174.67(8)	179.31(6)	179.16(5)	169.56(7)	169.9(1)
N2 Fe1 N3	78.07(8)	85.16(7)	85.06(5)	78.24(7)	78.3(1)
N2 Fe1 N4	106.17(8)	95.24(6)	95.22(5)	109.02(7)	108.8(1)
N3 Fe1 N4	77.24(8)	84.95(6)	84.70(5)	76.80(7)	76.8(1)
*α*	94.39(6)	75.84(4)	75.24(3)	106.12(5)	103.6(1)
angle (i-ii)	88.1(1)	31.2(1)	31.7(1)	85.7(1)	86.0(3)
angle (ii-iii)	84.3(1)	30.5(1)	31.0(1)	80.4(1)	81.2(2)
angle (i-iii)	35.4(1)	25.5(1)	25.1(1)	38.3(1)	38.0(3)

**Table 2:** Crystal structure and refinement data.

	1	2	2	3	3
Chemical formula	C_28_H_28_FeN_4_NiO_4_S_10_	C_22_H_30_FeN_5_O_8_	C_22_H_30_FeN_5_O_8_	C_22_H_28_FeIN_4_O_4_	C_22_H_28_FeIN_4_O_4_
Formula weight	919.70	548.36	548.36	595.23	595.23
Temperature (K)	120	120	293	100	293
Cell setting	triclinic	monoclinic	monoclinic	monoclinic	monoclinic
Space group, *Z*	*P*-1, 2	*P*2_1_/*c*, 4	*P*2_1_/*c*, 4	*P*2_1_/*n*, 4	*P*2_1_/*n*, 4
*a* (Å)	10.4149(8)	17.7659(8)	17.9547(3)	10.9107(1)	10.6616(3)
*b* (Å)	10.8546(9)	9.5962(4)	9. 6817(1)	17.223(1)	17.6910(3)
*c* (Å)	17.662(1)	14.4516(7)	14.4568(2)	13.7678(9)	13.4855(2)
*α* (^o^)	74.178(2)	90	90	90	90
*β* (^o^)	80.256(2)	110.495(1)	110.178(2)	106.136(1)	104.866(2)
*γ* (^o^)	68.484(1)	90	90	90	90
Cell volume (Å^3^)	1781.8(2)	2307.8(2)	2358.82(6)	2403.0(3)	2458.42(9)
Crystal size (mm)	0.22 × 0.15 × 0.14	0.29 × 0.26 × 0.13	0.60 × 0.19 × 0.05	0.45 × 0.43 × 0.35	0.57 × 0.36 × 0.15
ρ (Mg/m^3^)	1.714	1.578	1.544	1.645	1.608
μ (cm^−1^)	15.63	7.14	6.99	19.47	19.03
Refls collected/unique/observed with *I* > 2σ(*I*)	21631/9461/6661	27190/6138/5239	23118/6700/5748	18375/6360/5558	36353/8470/5887
*R*_int_	0.0399	0.0396	0.0242	0.0236	0.0260
θ_max_ (^o^)	29.00	29.00	31.01	29.00	32.71
Parameters refined	439	364	385	297	291
Final *R*_1_(obs), *wR*_2_ (all)	0.0365, 0.0732	0.0390, 0.0930	0.0310, 0.0870	0.0270, 0.0856	0.0568, 0.1758
Goodness-of-fit	0.999	1.058	1.055	1.014	1.078
Residual electron density (e Å^−3^)	0.461/−0.394	0.413/−0.549	0.372/−0.319	0.849/−0.829	2.364/−1.762
CCDC reference	2031156	2031157	2031158	2031159	2031160

## Supplementary Materials

The following are available online, Figure S1: Temperature dependence of the product, *I*_EPR_*T*, and the relative intensity value, *I*_EPR_, for the LS fraction *S* = 1/2 spin moments in **1**, Figure S2: Field dependences of the magnetization, *M*(*B/T*), for **1** (*T* = 2.0 K) for intermediate *T*-cycles (2, 3, 4—order of cycles), Figure S3: Temperature evolution of the EPR spectrum for **2**, Figure S4: Temperature evolution of the EPR spectrum for **3**, Figure S5: Temperature evolution components of *g*-tensor of the EPR spectrum: *g*_1_, *g*_2_, *g*_3_ for **2**, Figure S6: Temperature evolution components of *g*-tensor of the EPR spectrum: *g*_1_, *g*_2_, *g*_3_ for **3** (left scale) and linewidth, △*B*(*T*) (right scale), Figure S7: Total EPR spectrum of **1** at 100 K (low- and high-field segments) and respective simulations, Figure S8: Comparison of the EPR spectra in the vicinity of *g*~2.0 at 250 K for **1** at thermocycling. Blue line—original **1**, green line—after thermocyclings, red line—isostructural compound [Fe(III)(3-OMe-Sal_2_trien)] [Au(dmit)_2_] [10], Figure S9: Temperature dependence of the EPR linewidth, Δ*B*_2_^HS^, corresponding to the *g*_2_^HS^ of the HS phase of **1**. Figure S10: Photos single crystals of [Fe(3-OMe-Sal_2_trien)][Ni(dmit)_2_], Figure S11: Experimental powder X-ray diffraction pattern of [Fe(3-OMe-Sal_2_trien)][Ni(dmit)_2_] (**1**, solid) at room temperature compared with the simulated pattern from the single crystal X-ray data at 297 K (dots), Figure S12: Experimental powder X-ray diffraction pattern of [Fe(3-OMe-Sal_2_trien)]NO_3_·H_2_O (**2**, solid) at room temperature compared with the simulated pattern from the single crystal X-ray data at 297 K (dots) Figure S13: Analysis for a full-profile refinement using the Rietveld method, which made it possible to confirm not only the lattice parameters, but also the structure, was made. The experimental powder X-ray diffraction pattern of [Fe(3-OMe-Sal_2_trien)]I (**3**, blue line) at room temperature, red—calculated, gray—difference curve, Figure S14: Grinded polycrystals of **1** (**a**) and **2** (**b**) used in SQUID and EPR measurements. Pure green color of the transparent particles confirms LS ground state of **2**. Violet and brownish tones for **1** confirm presence of the HS phase in the ground state, Figure S14c: Grinded polycrystals of **3** used in SQUID and EPR measurements. Pure green color of the transparent particles confirms LS ground state of **3**, Figure S15: Optical microscopy images of the polycrystalline product of **3**: (**a**) Single crystals selected for SCXRD, (**b**) part of the polycrystalline sample selected for SQUID and EPR experiments, Figure S15c: Image of the sample **3** for RSMA, Figure S16: The thermograms of [Fe(3-OMe-Sal_2_trien)](NO_3_)•H_2_O (**2** H_2_O) (a), [Fe(3-OMe-Sal_2_trien)]I (**3**) (b) and [Fe(3-OMe-Sal_2_trien)][Ni(dmit)_2_] (**1**) (c), This material is available free of charge via the Internet at http://pubs.acs.org.
