# Supplementary material for: Magnetism, Conductivity and Spin-Spin Interactions in Layered Hybrid Structure of Anionic Radicals [Ni(dmit)2] Alternated by Iron(III) Spin-Crossover Complex [Fe(III)(3-OMe-Sal2trien)] and Ferric Moiety Precursors
Source: Molecules. 2020 Oct 24;25(21):4922. doi: 10.3390/molecules25214922 (PMC7663777; doi:10.3390/molecules25214922)
Supplement: Supplementary file 1 [file molecules-25-04922-s001.zip › Supplementary-materials_for_Molecules.pdf]

Electronic supporting information

Magnetism, Conductivity and Spin-Spin Interactions in Layered Hybrid Structure of Anionic Radicals [Ni(dmit)$_2$] Alternated by Iron(III) Spin-Crossover Complex [Fe(III)(3-OMe-Sal$_2$trien)] and Ferric Moiety Precursors.

Yuri N. Shvachko [1*], Nataliya G. Spitsyna [2], Denis V. Starichenko [1], Vladimir N. Zverev [3], Leokadiya V. Zorina[3], Sergey V. Simonov [3], Maksim A. Blagov [2,4], Eduard B. Yagubskii [2*]

[1] M.N. Miheev Institute of Metal Physics UB RAS, 620108 Yekaterinburg, Russia

[2] Institute of Problems of Chemical Physics RAS, 142432 Chernogolovka MD, Russia

[3] Institute of Solid State Physics RAS, 143432 Chernogolovka MD, Russia

[4] Lomonosov Moscow State University, 119991 Moscow, Russia

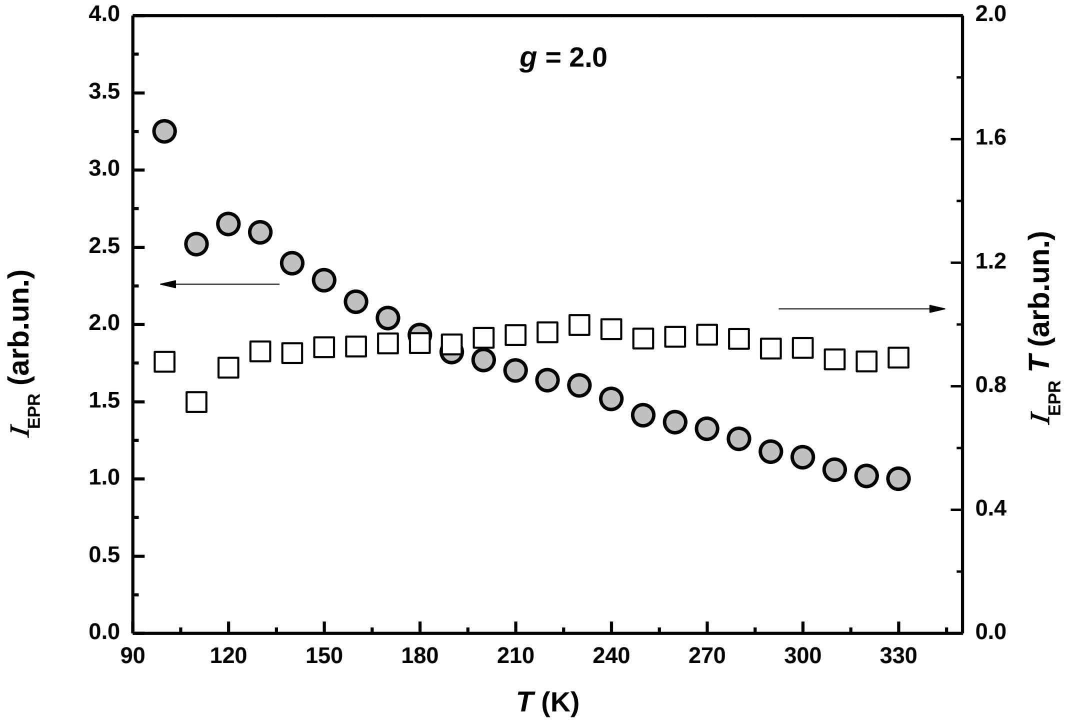

Figure S1. Temperature dependence of the product, $I_{EPR}T$, and the relative intensity value, I_{EPR}, for the LS fraction S = 1/2 spin moments in **1**.

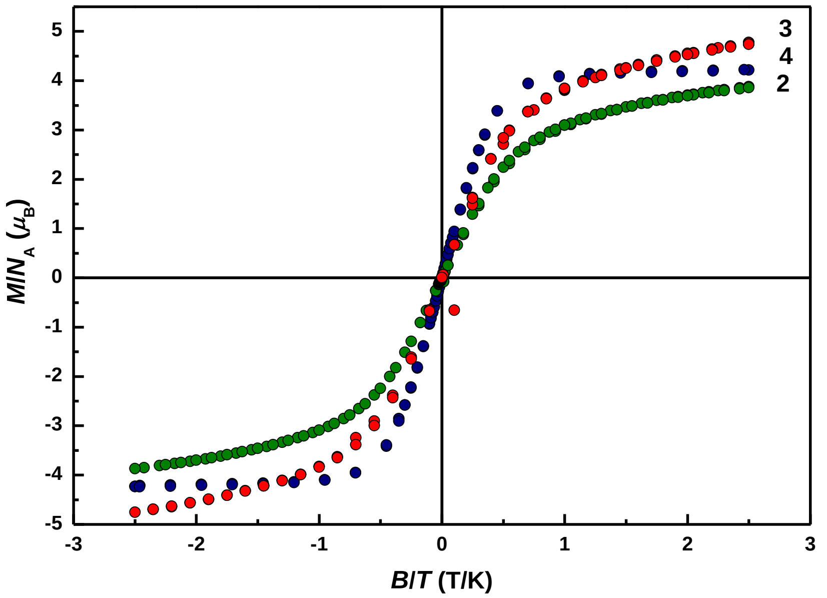

Figure S2. Field dependences of the magnetization, $M(B/T)$, for **1** (T = 2.0 K) for intermediate T-cycles (2, 3, 4 – order of cycles).

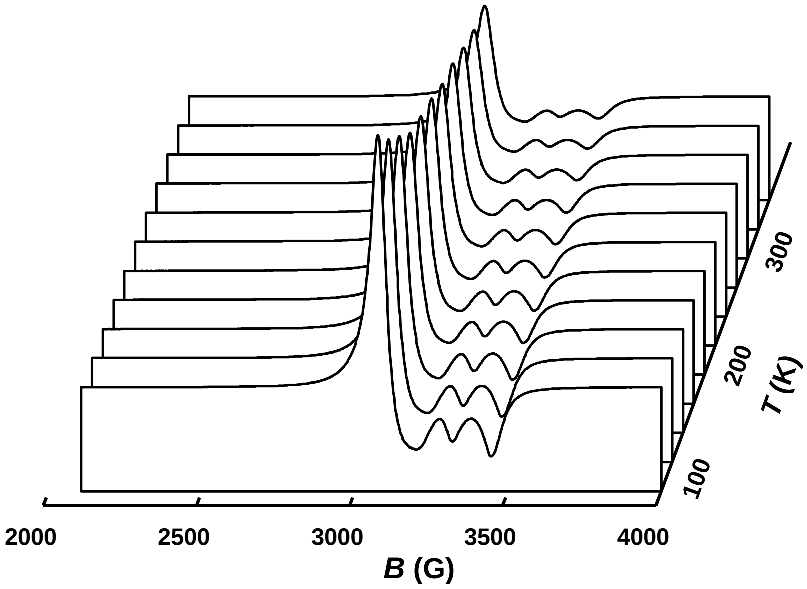

Figure S3. Temperature evolution of the EPR spectrum for **2**.

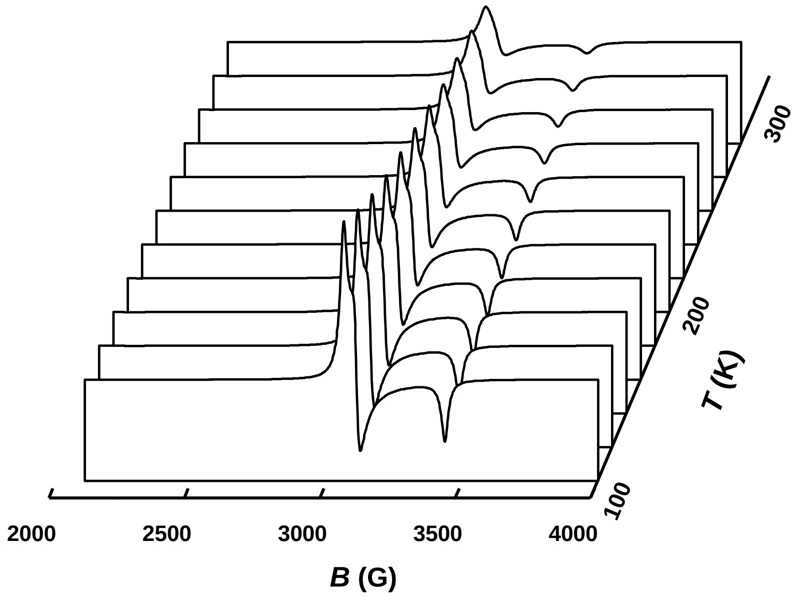

Figure S4. Temperature evolution of the EPR spectrum for **3**.

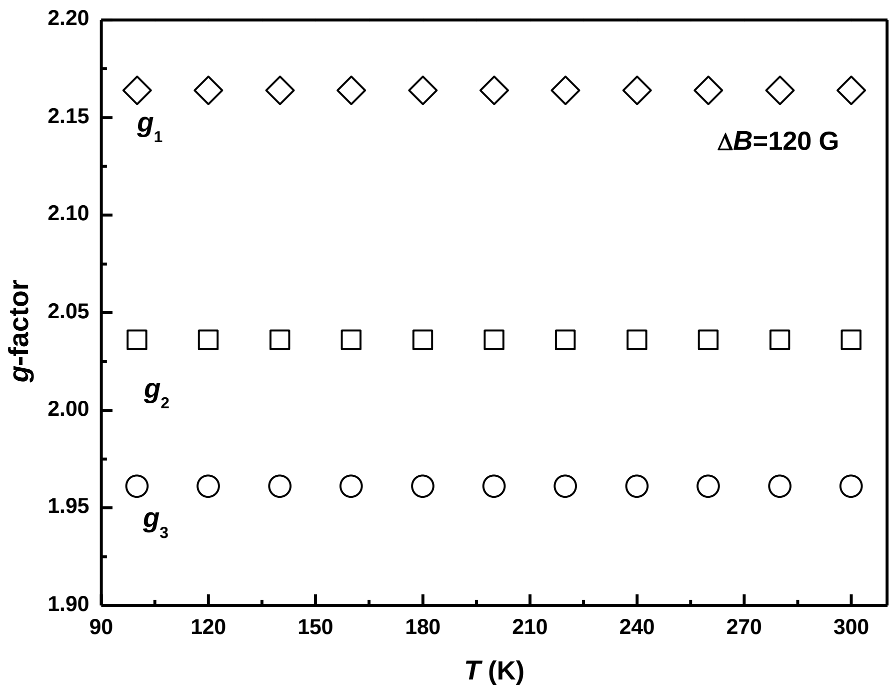

Figure S5. Temperature evolution components of *g*-tensor of the EPR spectrum: g_1, g_2, g_3 for **2**.

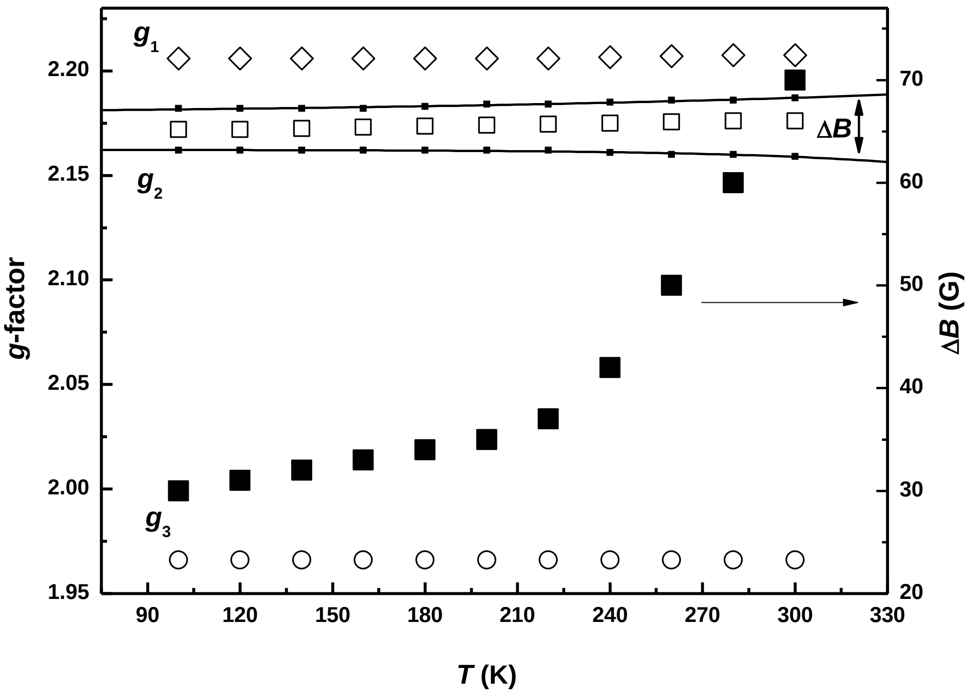

Figure S6. Temperature evolution components of *g*-tensor of the EPR spectrum: g_1, g_2, g_3 for **3** (left scale) and linewidth, $\triangle B(T)$ (right scale).

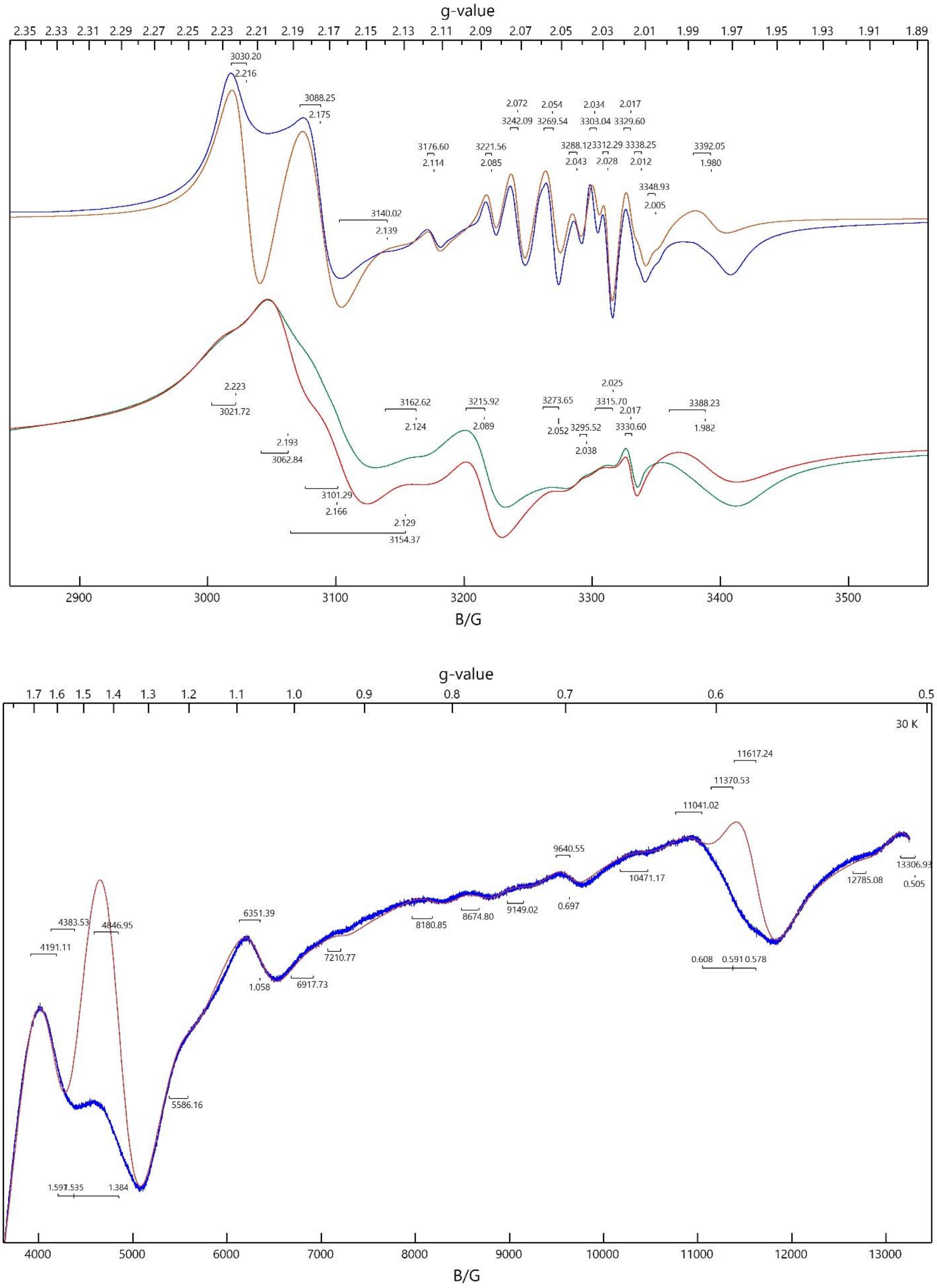

Figure S7. Total EPR spectrum of **1** at 100 K (low- and high-field segments) and respective simulations

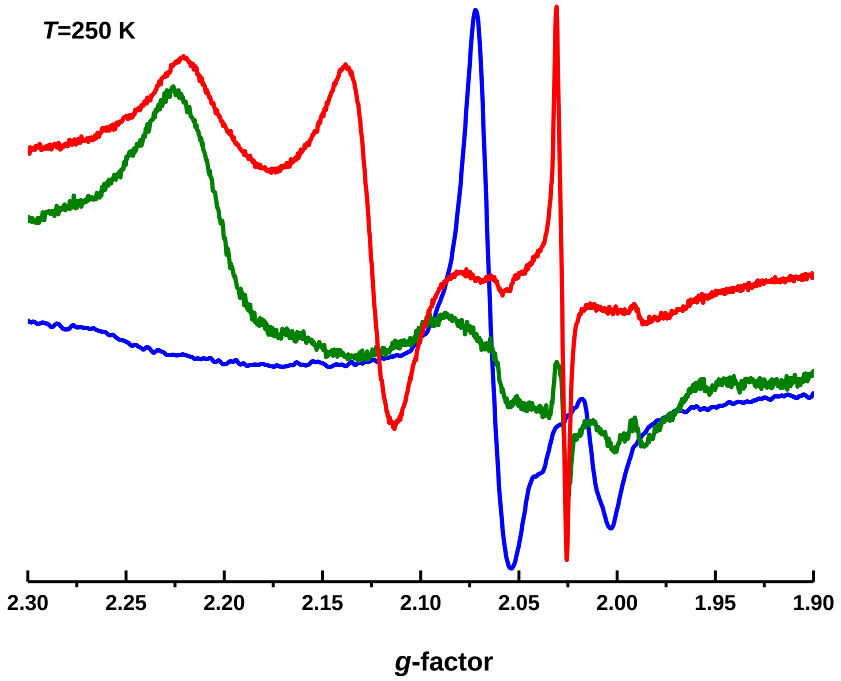

Figure S8. Comparison of the EPR spectra in the vicinity of g ~ 2.0 at 250 K for **1** at thermocycling. Blue line – original **1**, green line – after thermocyclings, red line – isostructural compound [Fe(III)(3-OMe-Sal_2trien)] [Au(dmit)$_2$] [10].

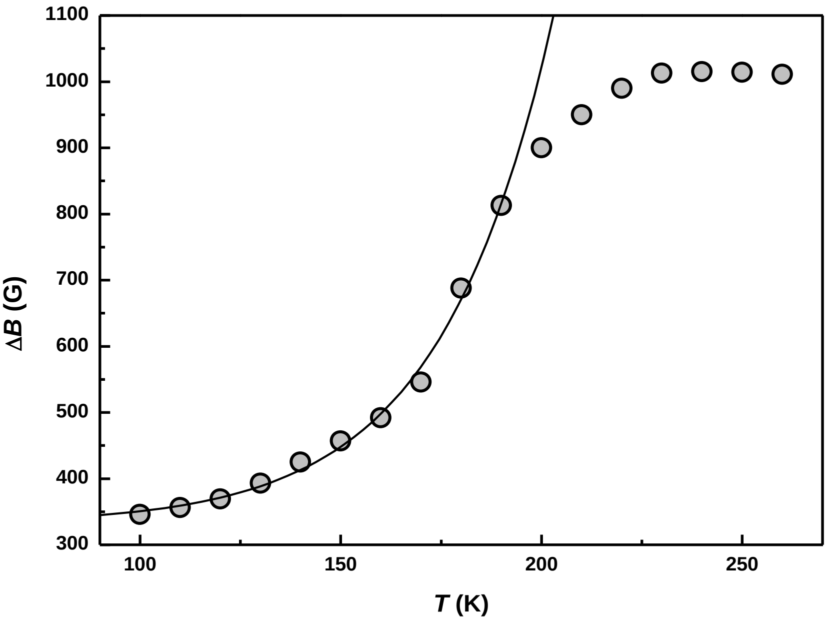

Figure S9. Temperature dependence of the EPR linewidth, ΔB_2^{HS}, corresponding to the g_2^{HS} of the HS phase of **1**.

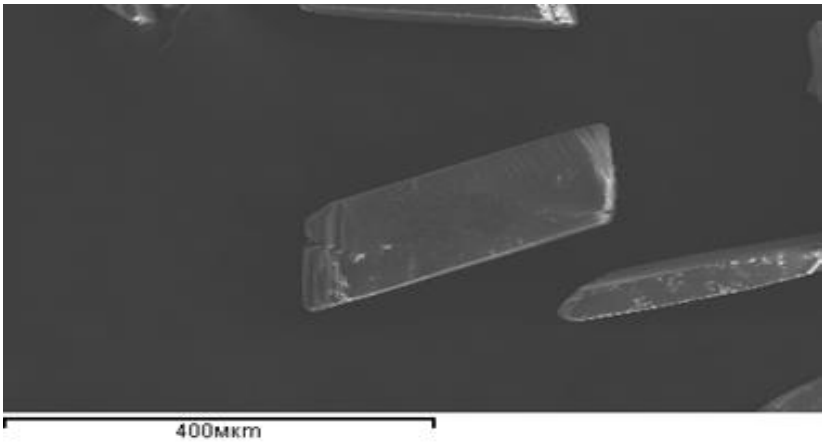

a)

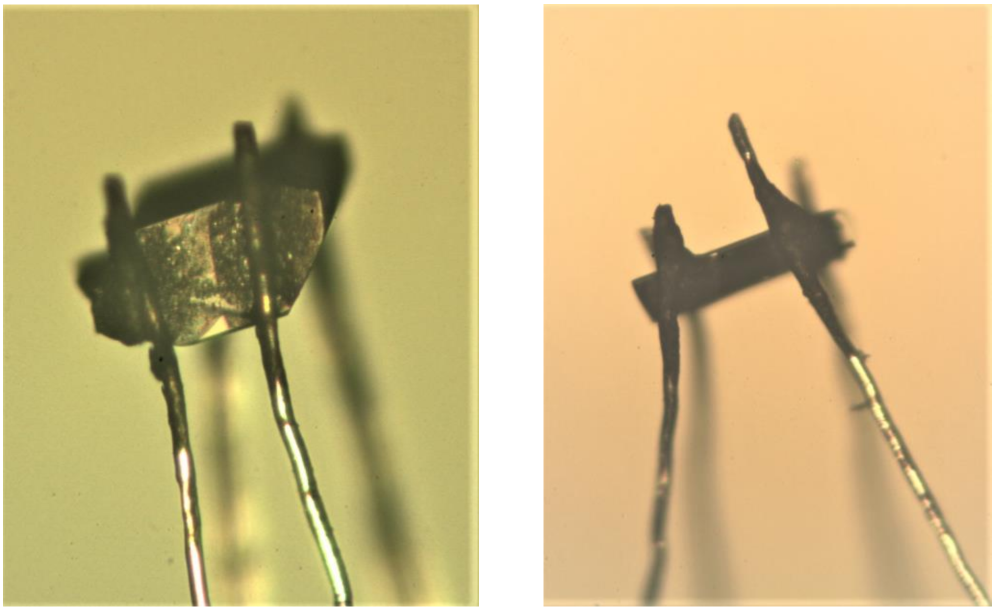

b)

Figure S10. Photos single crystals of [Fe(3-OMe-Sal$_2$trien)][Ni(dmit)$_2$] (**1**): immediately synthesized (upper photo **a**); during resistance measurements $\boldsymbol{J} \parallel \boldsymbol{c}$ ("transverse"), and $\boldsymbol{J} \perp \boldsymbol{c}$ ("in-plane") - left and right panels photo **b**, respectively.

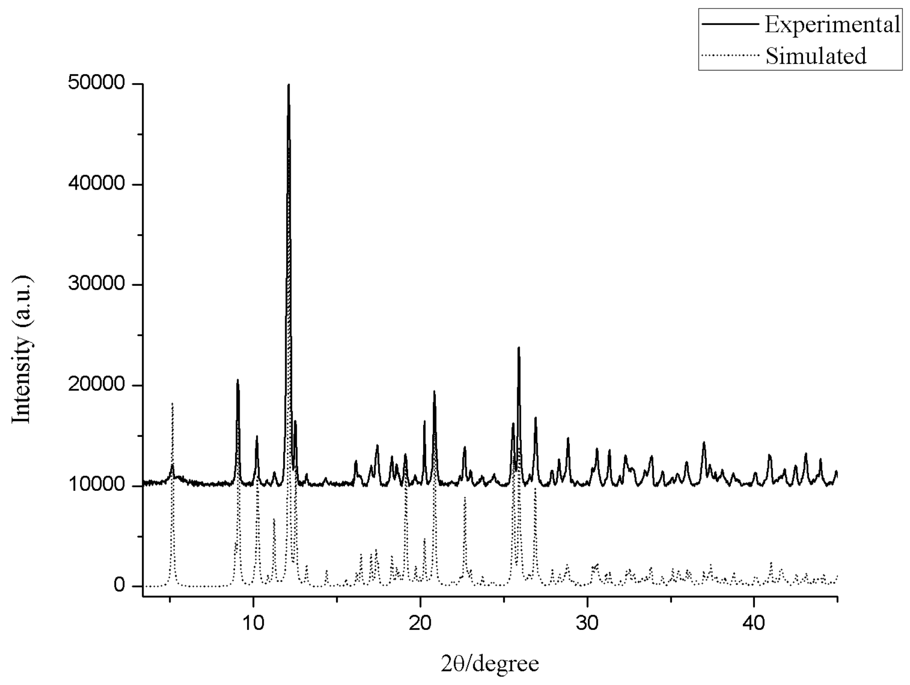

Figure S11. Experimental powder X-ray diffraction pattern of [Fe(3-OMe-Sal$_2$trien)][Ni(dmit)$_2$] (**1**, solid) at room temperature compared with the simulated pattern from the single crystal X-ray data at 297 K (dots).

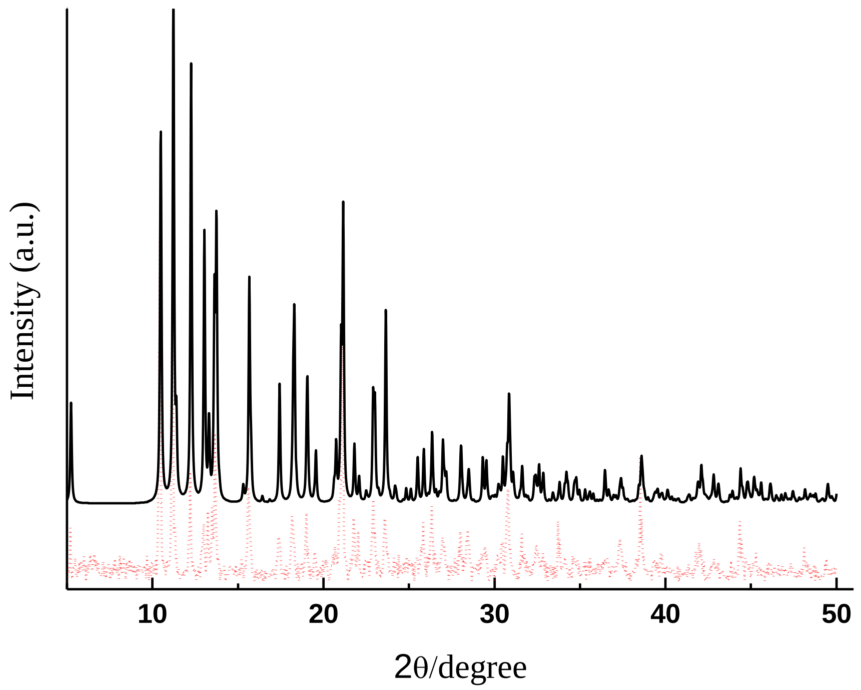

Figure S12. Experimental powder X-ray diffraction pattern of [Fe(3-OMe-Sal$_2$trien)]$NO_3 \cdot H_2O$ (**2**, solid) at room temperature compared with the simulated pattern from the single crystal X-ray data at 297 K (dots).

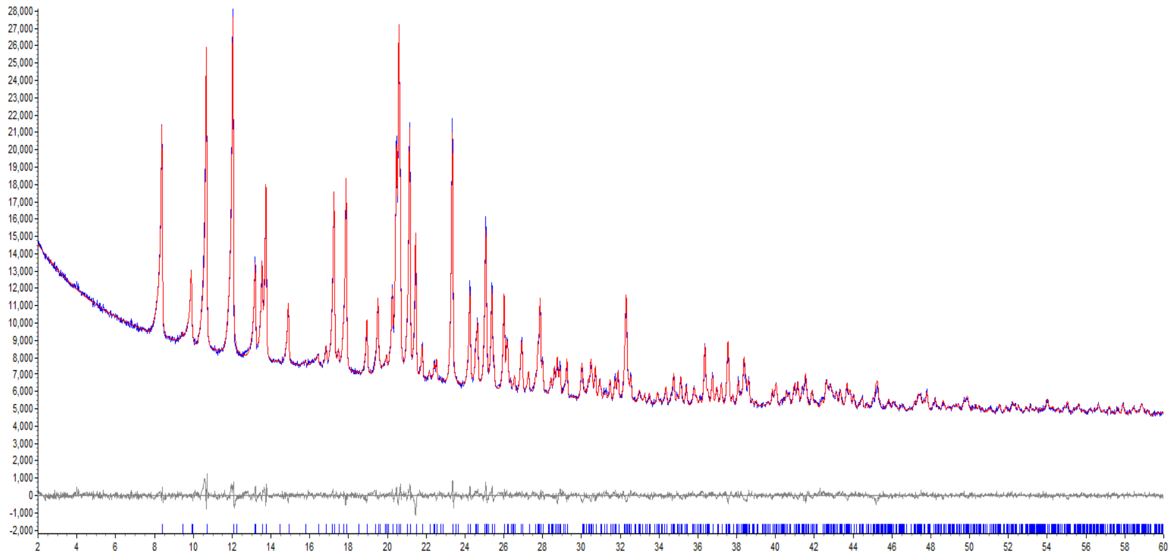

Figure S13. Analysis for a full-profile refinement using the Rietveld method, which made it possible to confirm not only the lattice parameters, but also the structure, was made. The experimental powder X-ray diffraction pattern of [Fe(3-OMe-Sal_2trien)]I (**3**, blue line) at room temperature, red - calculated, gray - difference curve.

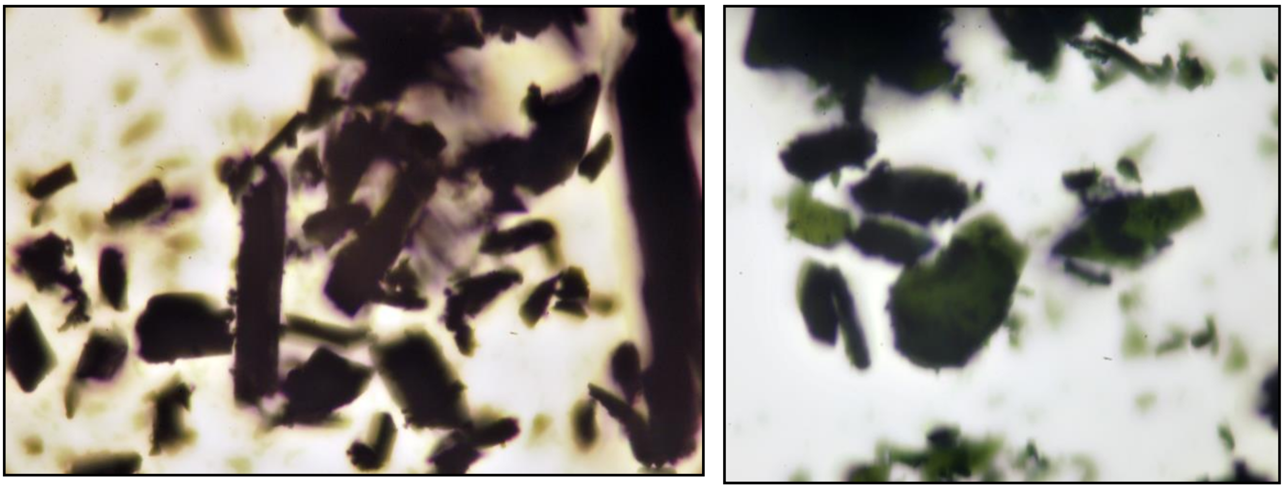

(a) **(b)**

Figure S14. Grinded polycrystals of **1** **(a)** and **2** **(b)** used in SQUID and EPR measurements. Pure green color of the transparent particles confirms LS ground state of **2**. Violet and brownish tones for **1** confirm presence of the HS phase in the ground state.

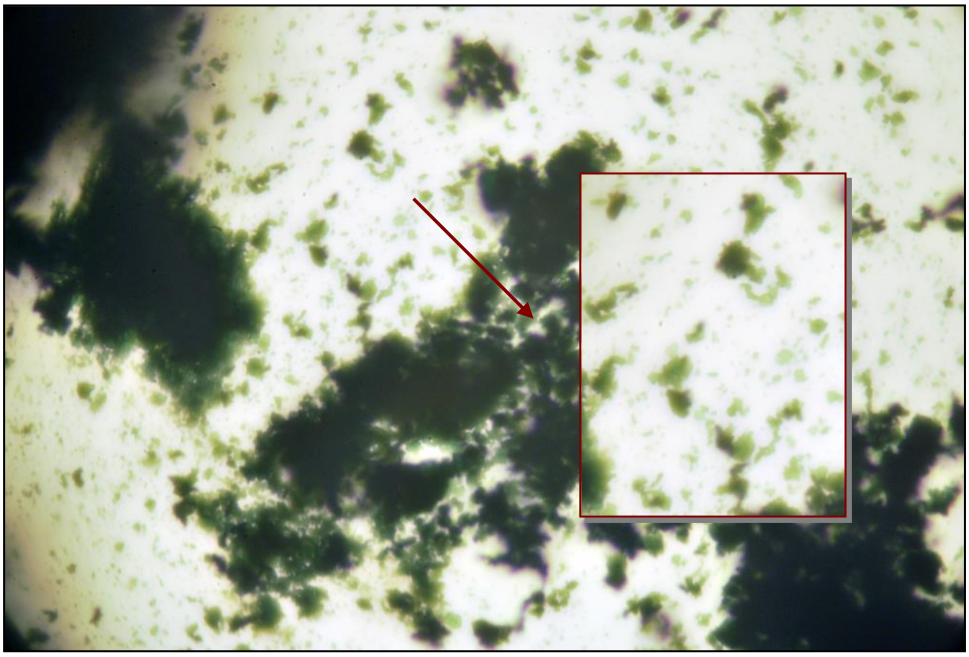

Figure S14c. Grinded polycrystals of **3** used in SQUID and EPR measurements. Pure green color of the transparent particles confirms LS ground state of **3**.

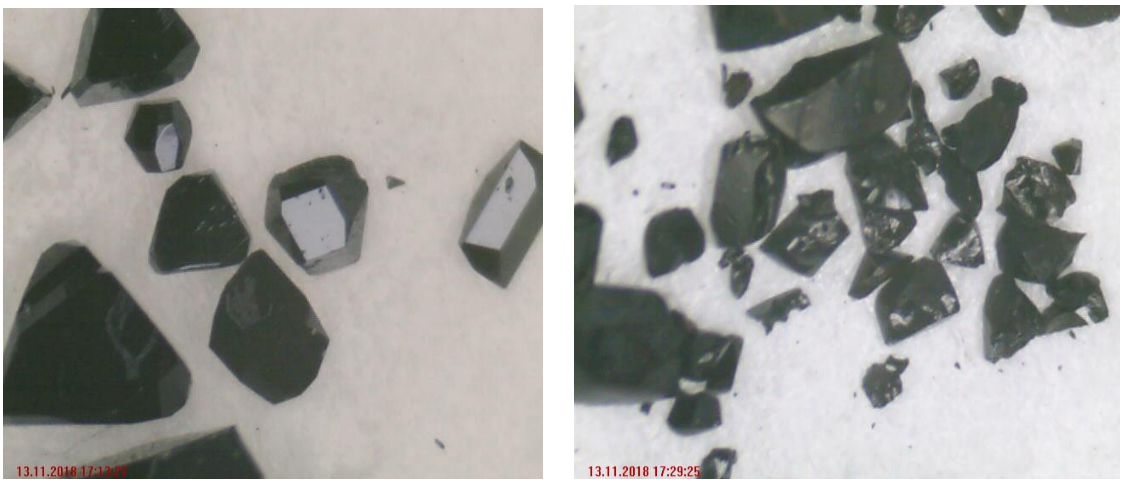

(a) **(b)**

Figure S15. Optical microscopy images of the polycrystalline product of **3**: **(a)** Single crystals selected for SCXRD, **(b)** part of the polycrystalline sample selected for SQUID and EPR experiments.

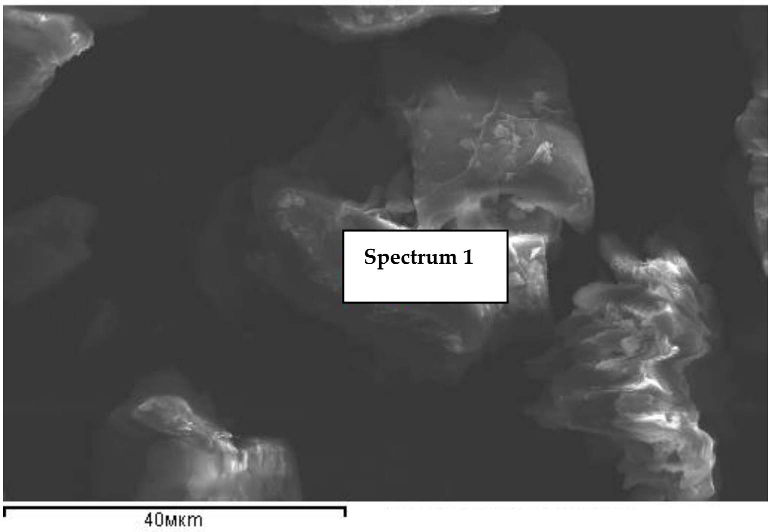

Figure S15c. Image of the sample **3** for RSMA.

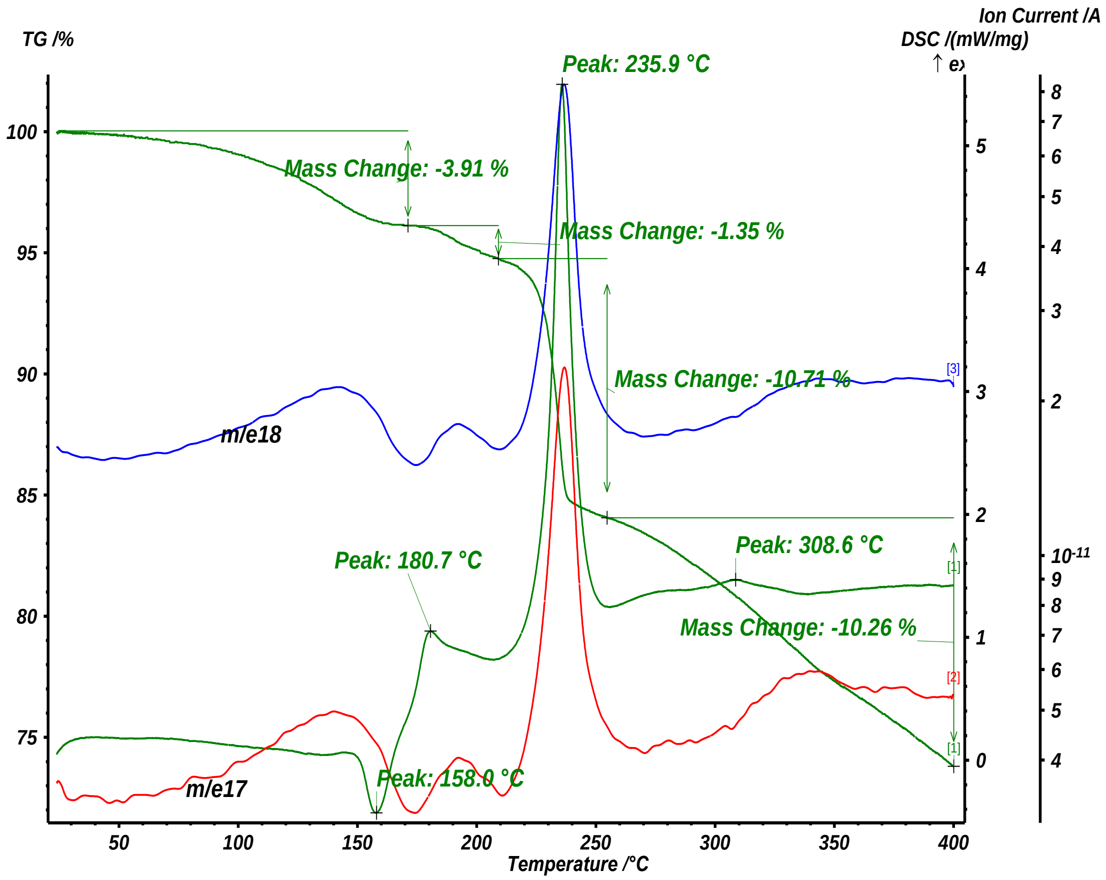

a

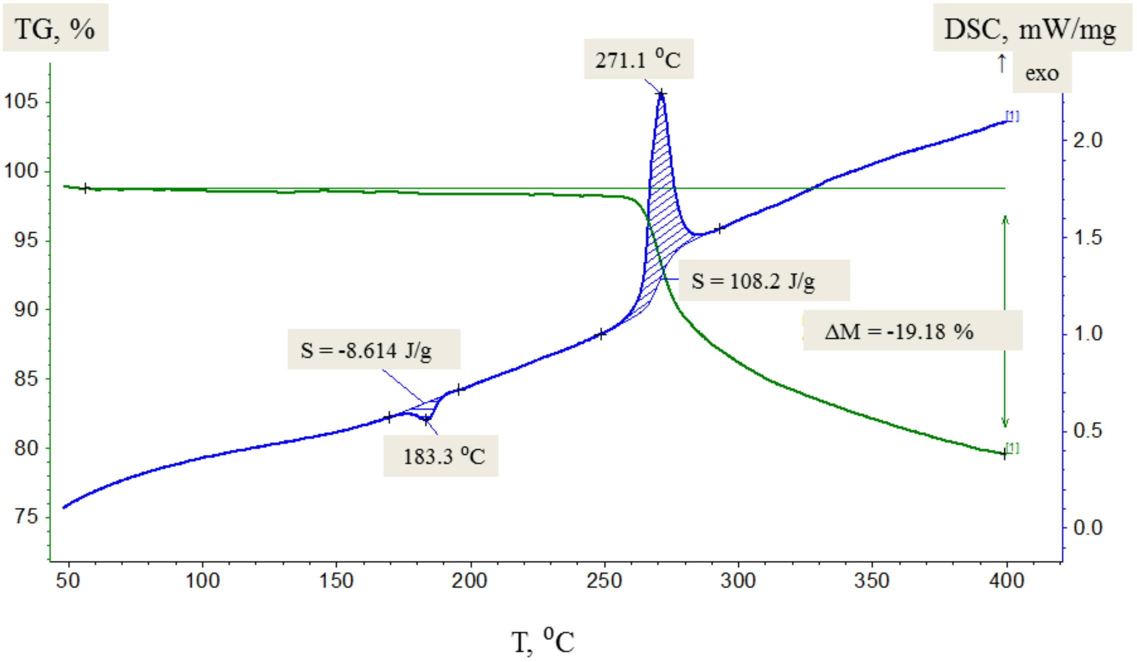

b

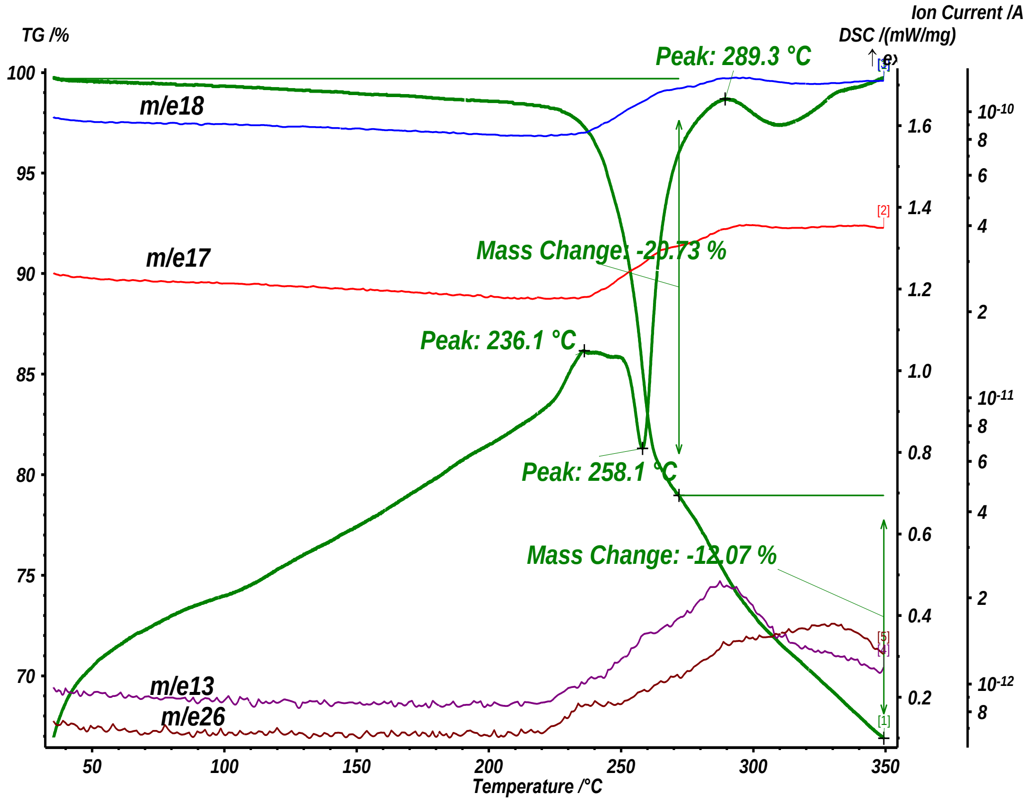

c

Figure S16. The thermograms of [Fe(3-OMe-Sal$_2$trien)](NO$_3$)•H$_2$O (**2**•H$_2$O) (a), [Fe(3-OMe-Sal$_2$trien)]I (**3**) (b) and [Fe(3-OMe-Sal$_2$trien)][Ni(dmit)$_2$] (**1**) (c).
