# Supplementary material for: Magnetism, Conductivity and Spin-Spin Interactions in Layered Hybrid Structure of Anionic Radicals [Ni(dmit)2] Alternated by Iron(III) Spin-Crossover Complex [Fe(III)(3-OMe-Sal2trien)] and Ferric Moiety Precursors
Source: Molecules. 2020 Oct 24;25(21):4922. doi: 10.3390/molecules25214922 (PMC7663777; doi:10.3390/molecules25214922)

checkCIF/PLATON report

You have not supplied any structure factors. As a result the full set of tests cannot be run.

THIS REPORT IS FOR GUIDANCE ONLY. IF USED AS PART OF A REVIEW PROCEDURE FOR PUBLICATION, IT SHOULD NOT REPLACE THE EXPERTISE OF AN EXPERIENCED CRYSTALLOGRAPHIC REFEREE.

No syntax errors found.   CIF dictionary   Interpreting this report

Datablock: 1-120K

```
Bond precision:  C-C = 0.0040 A          Wavelength=0.71073

Cell:          a=10.4149(8)       b=10.8546(9)      c=17.6618(14)
               alpha=74.1779(16)  beta=80.2564(16)  gamma=68.4839(15)
Temperature:  120 K
```

	Calculated	Reported
Volume	1781.8(2)	1781.8(2)
Space group	P -1	P -1
Hall group	-P 1	-P 1
Moiety formula	C22 H28 Fe N4 O4, C6 Ni S10	C22 H28 Fe N4 O4, C6 Ni S10
Sum formula	C28 H28 Fe N4 Ni O4 S10	C28 H28 Fe N4 Ni O4 S10
Mr	919.68	919.70
Dx,g cm-3	1.714	1.714
Z	2	2
Mu (mm-1)	1.563	1.563
F000	940.0	940.0
F000'	944.06	
h,k,lmax	14,14,24	14,14,24
Nref	9475	9461
Tmin,Tmax	0.755,0.803	0.725,0.811
Tmin'	0.709	

```
Correction method= # Reported T Limits: Tmin=0.725 Tmax=0.811
AbsCorr = MULTI-SCAN

Data completeness= 0.999          Theta(max)= 29.000

R(reflections)= 0.0365( 6661)     wR2(reflections)= 0.0732( 9461)

S = 0.999                   Npar= 439
```

The following ALERTS were generated. Each ALERT has the format
test-name_ALERT_alert-type_alert-level.
Click on the hyperlinks for more details of the test.

Alert level C

```
PLAT420_ALERT_2_C D-H Without Acceptor       N3       --H3N      .     Please Check
```

Alert level G

```
PLAT005_ALERT_5_G No Embedded Refinement Details Found  in the CIF     Please Do !
PLAT007_ALERT_5_G Number of Unrefined Donor-H Atoms .............          1 Report
PLAT232_ALERT_2_G Hirshfeld Test Diff (M-X)  Ni1      --S2       .        6.3 s.u.
PLAT710_ALERT_4_G Delete 1-2-3 or 2-3-4 Linear Torsion Angle ... #         1 Do !
              S1  -NI1 -S1A -C1SA   58.10  1.60   1.555   1.555   1.555   1.555
PLAT710_ALERT_4_G Delete 1-2-3 or 2-3-4 Linear Torsion Angle ... #         4 Do !
              S1A -NI1 -S1  -C1S   118.40  1.50   1.555   1.555   1.555   1.555
PLAT710_ALERT_4_G Delete 1-2-3 or 2-3-4 Linear Torsion Angle ... #         9 Do !
              S2A -NI1 -S2  -C2S   -45.80  0.80   1.555   1.555   1.555   1.555
PLAT710_ALERT_4_G Delete 1-2-3 or 2-3-4 Linear Torsion Angle ... #        12 Do !
              S2  -NI1 -S2A -C2SA -129.70  0.80   1.555   1.555   1.555   1.555
PLAT710_ALERT_4_G Delete 1-2-3 or 2-3-4 Linear Torsion Angle ... #        57 Do !
              N4  -FE1 -N1  -C7    -33.50  1.00   1.555   1.555   1.555   1.555
PLAT710_ALERT_4_G Delete 1-2-3 or 2-3-4 Linear Torsion Angle ... #        62 Do !
              N4  -FE1 -N1  -C8    144.70  0.80   1.555   1.555   1.555   1.555
PLAT710_ALERT_4_G Delete 1-2-3 or 2-3-4 Linear Torsion Angle ... #        87 Do !
              N1  -FE1 -N4  -C14   121.50  0.80   1.555   1.555   1.555   1.555
PLAT710_ALERT_4_G Delete 1-2-3 or 2-3-4 Linear Torsion Angle ... #        92 Do !
              N1  -FE1 -N4  -C13   -52.50  1.00   1.555   1.555   1.555   1.555
PLAT720_ALERT_4_G Number of Unusual/Non-Standard Labels ..........          3 Note
PLAT793_ALERT_4_G Model has Chirality at N2          (Centro SPGR)          R Verify
PLAT793_ALERT_4_G Model has Chirality at N3          (Centro SPGR)          S Verify
PLAT794_ALERT_5_G Tentative Bond Valency for Ni1       (III)     .       2.84 Info
PLAT794_ALERT_5_G Tentative Bond Valency for Fe1       (III)     .       2.99 Info
PLAT899_ALERT_4_G SHELXL97  is Deprecated and Succeeded by SHELXL/       2018 Note
```

---

0 **ALERT level A** = Most likely a serious problem - resolve or explain
0 **ALERT level B** = A potentially serious problem, consider carefully
1 **ALERT level C** = Check. Ensure it is not caused by an omission or oversight
17 **ALERT level G** = General information/check it is not something unexpected

0 ALERT type 1 CIF construction/syntax error, inconsistent or missing data
2 ALERT type 2 Indicator that the structure model may be wrong or deficient
0 ALERT type 3 Indicator that the structure quality may be low
12 ALERT type 4 Improvement, methodology, query or suggestion
4 ALERT type 5 Informative message, check

It is advisable to attempt to resolve as many as possible of the alerts in all categories. Often the minor alerts point to easily fixed oversights, errors and omissions in your CIF or refinement strategy, so attention to these fine details can be worthwhile. In order to resolve some of the more serious problems it may be necessary to carry out additional measurements or structure refinements. However, the purpose of your study may justify the reported deviations and the more serious of these should normally be commented upon in the discussion or experimental section of a paper or in the "special_details" fields of the CIF. checkCIF was carefully designed to identify outliers and unusual parameters, but every test has its limitations and alerts that are not important in a particular case may appear. Conversely, the absence of alerts does not guarantee there are no aspects of the results needing attention. It is up to the individual to critically assess their own results and, if necessary, seek expert advice.

Publication of your CIF in IUCr journals

A basic structural check has been run on your CIF. These basic checks will be run on all CIFs submitted for publication in IUCr journals (*Acta Crystallographica*, *Journal of Applied Crystallography*, *Journal of Synchrotron Radiation*); however, if you intend to submit to *Acta Crystallographica Section C* or *E* or *IUCrData*, you should make sure that full publication checks are run on the final version of your CIF prior to submission.

Publication of your CIF in other journals

Please refer to the *Notes for Authors* of the relevant journal for any special instructions relating to CIF submission.

PLATON version of 10/08/2020; check.def file version of 06/08/2020

Datablock 1-120K - ellipsoid plot

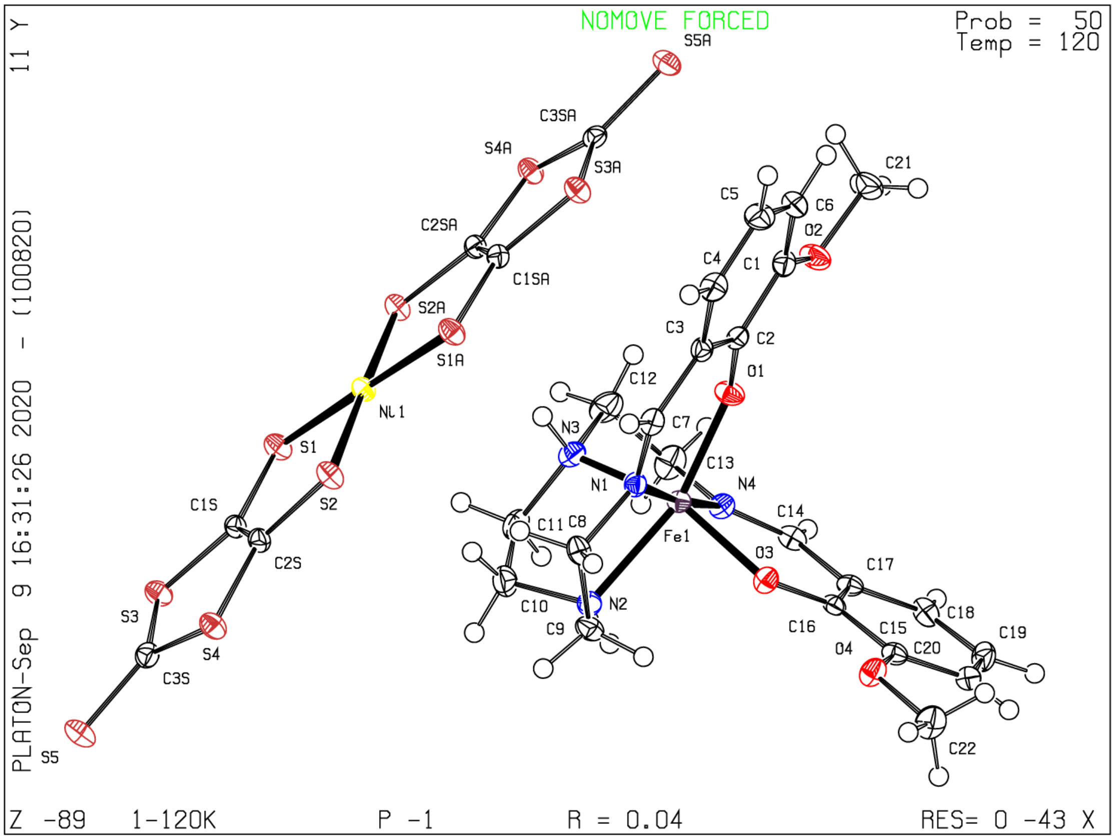

Supplement: Supplementary file 1 [file molecules-25-04922-s001.zip › 1_120K_checkcif.pdf]
