# Supplementary material for: Magnetism, Conductivity and Spin-Spin Interactions in Layered Hybrid Structure of Anionic Radicals [Ni(dmit)2] Alternated by Iron(III) Spin-Crossover Complex [Fe(III)(3-OMe-Sal2trien)] and Ferric Moiety Precursors
Source: Molecules. 2020 Oct 24;25(21):4922. doi: 10.3390/molecules25214922 (PMC7663777; doi:10.3390/molecules25214922)

checkCIF/PLATON report

You have not supplied any structure factors. As a result the full set of tests cannot be run.

THIS REPORT IS FOR GUIDANCE ONLY. IF USED AS PART OF A REVIEW PROCEDURE FOR PUBLICATION, IT SHOULD NOT REPLACE THE EXPERTISE OF AN EXPERIENCED CRYSTALLOGRAPHIC REFEREE.

No syntax errors found.   CIF dictionary   Interpreting this report

Datablock: 3-293K

```
Bond precision:  C-C = 0.0061 A          Wavelength=0.71073

Cell:            a=10.6616(3)    b=17.6910(3)       c=13.4855(2)
                 alpha=90        beta=104.866(2)    gamma=90
Temperature:     293 K

                Calculated                  Reported
Volume          2458.42(9)                  2458.42(9)
Space group     P 21/n                      P 21/n
Hall group      -P 2yn                      -P 2yn
Moiety formula  C22 H28 Fe N4 O4, I         ?
Sum formula     C22 H28 Fe I N4 O4          C22 H28 Fe I N4 O4
Mr              595.23                      595.23
Dx,g cm-3       1.608                       1.608
Z               4                           4
Mu (mm-1)       1.903                       1.903
F000            1196.0                      1196.0
F000'           1195.89
h,k,lmax        16,26,20                    16,26,20
Nref            9065                        8470
Tmin,Tmax       0.439,0.752                 0.689,1.000
Tmin'           0.335

Correction method= # Reported T Limits: Tmin=0.689 Tmax=1.000
AbsCorr = MULTI-SCAN

Data completeness= 0.934          Theta(max)= 32.714

R(reflections)= 0.0568( 5887)     wR2(reflections)= 0.1758( 8470)

S = 1.078                  Npar= 291
```

The following ALERTS were generated. Each ALERT has the format
test-name_ALERT_alert-type_alert-level.
Click on the hyperlinks for more details of the test.

Alert level B

```
PLAT005_ALERT_5_B No Embedded Refinement Details Found  in the CIF     Please Do !
```

Alert level C

```
PLAT220_ALERT_2_C NonSolvent   Resd 1  C   Ueq(max)/Ueq(min) Range        3.4 Ratio
PLAT241_ALERT_2_C High   'MainMol' Ueq as Compared to Neighbors of         C5 Check
```

Alert level G

```
PLAT007_ALERT_5_G Number of Unrefined Donor-H Atoms ..............          2 Report
PLAT199_ALERT_1_G Reported _cell_measurement_temperature ..... (K)        293 Check
PLAT200_ALERT_1_G Reported   _diffrn_ambient_temperature ..... (K)        293 Check
PLAT793_ALERT_4_G Model has Chirality at N2          (Centro SPGR)          S Verify
PLAT793_ALERT_4_G Model has Chirality at N3          (Centro SPGR)          R Verify
PLAT794_ALERT_5_G Tentative Bond Valency for Fe1       (III)     .       2.98 Info
PLAT883_ALERT_1_G No Info/Value for _atom_sites_solution_primary .     Please Do !
PLAT964_ALERT_2_G SHELXL WEIGHT Parameter in CIF & RES Differ ....     Please Check
PLAT965_ALERT_2_G The SHELXL WEIGHT Optimisation has not Converged     Please Check
```

0 **ALERT level A** = Most likely a serious problem - resolve or explain
1 **ALERT level B** = A potentially serious problem, consider carefully
2 **ALERT level C** = Check. Ensure it is not caused by an omission or oversight
9 **ALERT level G** = General information/check it is not something unexpected

3 ALERT type 1 CIF construction/syntax error, inconsistent or missing data
4 ALERT type 2 Indicator that the structure model may be wrong or deficient
0 ALERT type 3 Indicator that the structure quality may be low
2 ALERT type 4 Improvement, methodology, query or suggestion
3 ALERT type 5 Informative message, check

It is advisable to attempt to resolve as many as possible of the alerts in all categories. Often the minor alerts point to easily fixed oversights, errors and omissions in your CIF or refinement strategy, so attention to these fine details can be worthwhile. In order to resolve some of the more serious problems it may be necessary to carry out additional measurements or structure refinements. However, the purpose of your study may justify the reported deviations and the more serious of these should normally be commented upon in the discussion or experimental section of a paper or in the "special_details" fields of the CIF. checkCIF was carefully designed to identify outliers and unusual parameters, but every test has its limitations and alerts that are not important in a particular case may appear. Conversely, the absence of alerts does not guarantee there are no aspects of the results needing attention. It is up to the individual to critically assess their own results and, if necessary, seek expert advice.

Publication of your CIF in IUCr journals

A basic structural check has been run on your CIF. These basic checks will be run on all CIFs submitted for publication in IUCr journals (*Acta Crystallographica*, *Journal of Applied Crystallography*, *Journal of Synchrotron Radiation*); however, if you intend to submit to *Acta Crystallographica Section C* or *E* or *IUCrData*, you should make sure that full publication checks are run on the final version of your CIF prior to submission.

Publication of your CIF in other journals

Please refer to the *Notes for Authors* of the relevant journal for any special instructions relating to CIF submission.

PLATON version of 10/08/2020; check.def file version of 06/08/2020

Datablock 3-293K - ellipsoid plot

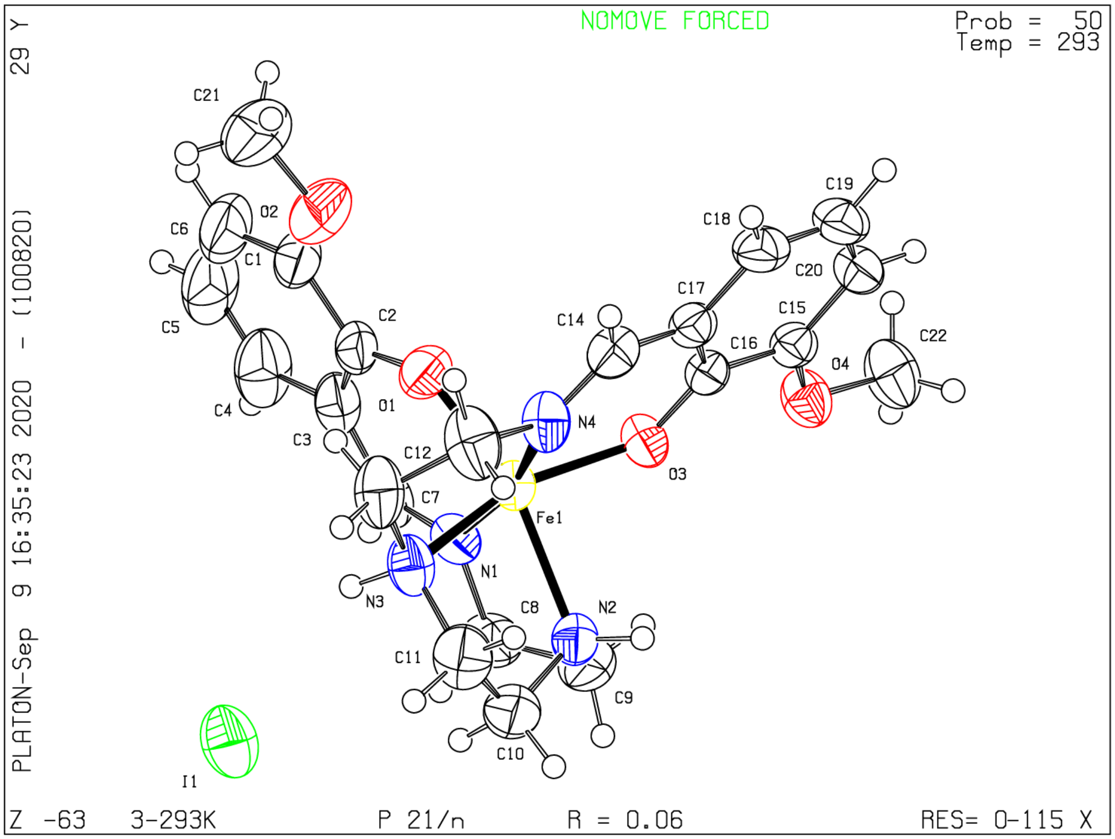

Supplement: Supplementary file 1 [file molecules-25-04922-s001.zip › 3_293K_checkcif.pdf]
